# Quality evaluation of compounds in leaves of six *Taxus* species based on UPLC-MS/MS and chemometrics

**DOI:** 10.3389/fchem.2023.1193188

**Published:** 2023-05-31

**Authors:** Qingzhu Cai, Qiang Song, Kunxia Jiang, Yao Lin, Ying Zhang, Jirong Zhang, Shuqing Lin, Lina Huang, Qihuang Xue, Zehao Huang, Wen Xu, Wei Xu, Mun Fei Yam

**Affiliations:** ^1^ College of Pharmacy, Fujian University of Traditional Chinese Medicine, Fuzhou, China; ^2^ Department of Pharmacy, Fujian Provincial Hospital, Shengli Clinical Medical College of Fujian Medical University, Fuzhou, China; ^3^ Fujian South Pharmaceutical Co., Ltd., Sanming, China; ^4^ Department of Pharmacology, School of Pharmaceutical Sciences, Universiti Sains Malaysia, Penang, Malaysia

**Keywords:** *Taxus* species, taxoids, flavonoids, chemometrics, quality control

## Abstract

**Introduction:** *Taxus* species are used as medicinal plants all over the world. The leaves of *Taxus* species are sustainable medicinal resources that are rich in taxoids and flavonoids. However, traditional identification methods cannot effectively identify *Taxus* species on the basis of leaces used as raw medicinal materials, because their appearance and morphological characteristics are almost the same, and the probability of error identification increases in accordance with the subjective consciousness of the experimenter. Moreover, although the leaves of different *Taxus* species have been widely used, their chemical components are similar and lack systematic comparative research. Such a situation is challenging for quality assessment.

**Materials and methods:** In this study, ultra-high-performance liquid chromatography coupled with triple quadrupole mass spectrometry combined with chemometrics was applied for the simultaneous determination of eight taxoids, four flavanols, five flavonols, two dihydroflavones, and five biflavones in the leaves of six *Taxus* species, namely, *T. mairei*, *T. chinensis*, *T. yunnanensis*, *T. wallichiana*, *T. cuspidata*, and *T. media*. Chemometric methods, including hierarchical cluster analysis, principal component analysis, orthogonal partial least squares-discriminate analysis, random forest iterative modeling, and fisher linear discriminant analysis, were utilized to differentiate and evaluate the six *Taxus* species.

**Results:** This proposed method exhibited good linearity (*R* ^2^ = 0.9999–0.9972) with a lower quantification limits of 0.94–3.05 ng/mL for all analytes. The intra- and inter-day precisions were within 6.83%. Six compounds, namely, 7-xylosyl-10-deacetyltaxol, ginkgetin, rutin, aromadendrin, 10-deacetyl baccatin III, and epigallocatechin, were identified through chemometrics for the first time. These compounds can be used as important chemical markers to distinguish the above six *Taxus* species rapidly.

**Conclusion:** This study established a method for determination of the leaves of six *Taxus* species, and revealing the differences in the chemical components of these six *Taxus* species.

## 1 Introduction

*Taxus* species, also called yew, are evergreen arbors or shrubs that belong to family Taxaceae and genus *Taxus* (Zhang et al., 2021). The taxoids extracted from *Taxus* species, such as taxol, 10-deacetyl baccatin III (10-DAB), and 10-deacetyltaxol (10-DAT), play an important role as precious medicinal plant resources in cancer treatment (Hafezi et al., 2020). Moreover, *Taxus* species are rich in flavonoids, such as sciadopitysin (SDN), quercitrin (QC), and ginkgetin (GK), which can inhibit tumor metastasis and treat osteoporosis, diabetic osteopathy, and Alzheimer’s disease (Gu et al., 2013; Weng et al., 2018). In the long run, the extensive use of the bark or root of *Taxus* species for the extraction of active substances will lead to the destruction of *Taxus* resources (Zhao et al., 2016). The leaves of *Taxus* species are also abundant in taxoids and flavonoids, which can replace the bark or roots of trees such that *Taxus* resources can be recycled (Yang et al., 2016). However, when the leaves of *Taxus* species are used as raw medicinal materials, identifying their varieties with subjective consciousness is difficult owing to their similar appearances. Among *Taxus* species, *T. chinensis*, *T. mairei*, *T. wallichiana*, and *T. cuspidata* are employed as traditional Chinese medicine, and *T. yunnanensis* and *T. media* are applied for the extraction of medicinal materials (Sharma and Garg, 2015). Thus, how to identify raw medicinal materials effectively has become a problem. At present, the leaves of different *Taxus* species are used as raw medicinal materials. However, the similarities in their chemical components have not been systematically compared. Therefore, we need to evaluate the differences in chemical components in the leaces of different *Taxus* varieties systematically and establish a chemical model to distinguish six different *Taxus* species on the basis of chemical content data of leaves to lay a foundation for the sustainable development and utilization of *Taxus*.

Huang et al. established a high-performance liquid chromatography (HPLC) method to determine five flavonoids in *T. mairei* (Huang et al., 2018). Cui et al. utilized HPLC to determine seven taxoids from *T. cuspidata*, *T. mairei*, and *T. media* (Cui et al., 2022). Li et al. applied HPLC coupled with tandem mass spectrometry (HPLC–MS/MS) to determine seven taxoids in *T. cuspidata*, *T. mairei*, and *T. media* (Li et al., 2009). The above methods require long analysis times but have and low detection sensitivity. Ultra–high–performance liquid chromatography (UPLC) has developed with the maturation of analytical technology. It has faster speed, higher efficiency, and higher sensitivity than HPLC. Moreover, UPLC–MS/MS has a good separation effect for complex multicomponent systems and can quickly and accurately quantify complex components (Tan et al., 2018; Xu and HeZhong, 2021). Wang et al. used UPLC–electrospray ionization (ESI)–MS/MS to analyze the metabolic changes in flavonoids in the leaves of *T. mairei* and *T. media* (Wang T. et al., 2019). Gai et al. used UPLC–MS/MS to determine the changes in the contents of seven taxoids and seven flavonoids in different parts of *T. cuspidata*, *T. mairei*, and *T. media* simultaneously (Gai et al., 2020). Supplementary Table S1 compares the results of our present study with those works in accordance with sample type, quantitative analytes, method time, mobile phase solvent consumption, limit of detection (LOD), and limit of quantification (LOQ). No research has been reported on the evaluation of the comprehensive quality of *Taxus* by UPLC–MS/MS combined with chemometrics.

In this study, UPLC–MS/MS combined with chemometrics was used for the first time to evaluate the comprehensive quality of the leaves of six *Taxus* species. A UPLC–MS/MS method was established for the simultaneous determination of 24 components, including eight taxoids, four flavanols, five flavonols, two dihydroflavones, and five biflavones, in the leaves of six *Taxus* species. The chemical components screened by chemometric methods, such as hierarchical cluster analysis (HCA), principal component analysis (PCA), orthogonal partial least squares-discriminate analysis (OPLS-DA), random forest (RF) iterative modeling, and fisher linear discriminant analysis (FDA), can provide references for the identification and quality evaluation of the above six different *Taxus* species.

## 2 Materials and methods

### 2.1 Samples

Fifty-one leaf samples were collected from six *Taxus* species in China and stored at 4°C (S1–15: *T. mairei*, S16–20: *T. chinensis*, S21–25: *T. yunnanensis*, S26–30: *T. wallichiana*, S31–36: *T. cuspidata*, S37–42: *T. media*, S43–51: for the external validation of FDA models). The images and detailed information of the samples are shown in Supplementary Figure S1 and Table 1. The sources of all samples were identified by Professor ZH and MY from the Plant Identification Teaching and Research Office of the Fujian University of Traditional Chinese Medicine, Fujian Province, China. Voucher specimens were kept in the Comprehensive Medical Research Institute of Fujian University of Traditional Chinese Medicine.

**TABLE 1:** Detailed information of 51 leaf samples from the six *Taxus* species.

Sample No	Specimen No	Variety	Source
S1	NH-1	*T. mairei*.	Mingxi, Fujian
S2	NH-2		Anxi, Fujian
S3	NH-3		Mingxi, Fujian
S4	NH-4		Mingxi, Fujian
S5	NH-5		Huzhou, Zhejiang
S6	NH-6		Yichun, Jiangxi
S7	NH-7		Minhou, Fujian
S8	NH-8		Minhou, Fujian
S9	NH-9		Mingxi, Fujian
S10	NH-10		Minqing, Fujian
S11	NH-11		Mingxi, Fujian
S12	NH-12		Ganzhou, Jiangxi
S13	NH-13		Yongzhou, Hunan
S14	NH-14		Zhangzhou, Fujian
S15	NH-15		Qiandongnan, Guizhou
S16	H-1	*T. chinensis.*	Tianshui, Gansu
S17	H-2		Weinan, Shanxi
S18	H-3		Weinan, Shanxi
S19	H-4		Weinan, Shanxi
S20	H-5		Tianshui, Gansu
S21	YH-1	*T. yunnanensis.*	Kunming, Yunnan
S22	YH-2		Kunming, Yunnan
S23	YH-3		Yongtai, Fujian
S24	YH-4		Yongtai, Fujian
S25	YH-5		Dali, Yunnan
S26	ZH-1	*T. wallichiana.*	Hami, Xinjiang
S27	ZH-2		Hami, Xinjiang
S28	ZH-3		Hami, Xinjiang
S29	ZH-4		Daqing, Heilongjiang
S30	ZH-5		Daqing, Heilongjiang
S31	DH-1	*T. cuspidata.*	Mianyang, Sichuan
S32	DH-2		Changchun, Jilin
S33	DH-3		Tonghua, Jilin
S34	DH-4		Anshan, Liaoning
S35	DH-5		Mudanjiang, Heilongjiang
S36	DH-6		Mudanjiang, Heilongjiang
S37	MH-1	*T. media.*	Wuxi, Jiangsu
S38	MH-2		Wuxi, Jiangsu
S39	MH-3		Linyi, Shandong
S40	MH-4		Xinyi, Jiangsu
S41	MH-5		Xinyi, Jiangsu
S42	MH-6		Chengdu, Sichuan
S43	NH-16	*T. mairei*.	Ninghua, Fujian
S44	NH-17		Yongtai, Fujian
S45	H-6	*T. chinensis.*	Wenxian, Gansu
S46	YH-6	*T. yunnanensis.*	Tengchong, Yunnan
S47	YH-7		Gongshan, Yunnan
S48	ZH-6	*T. wallichiana.*	Shannan, Xizang
S49	DH-7	*T. cuspidata.*	Fusong, Jilin
S50	DH-8		Fusong, Jilin
S51	MH-7	*T. media.*	Cangnan, Zhejiang

### 2.2 Reagents and standards

**FIGURE 1:**
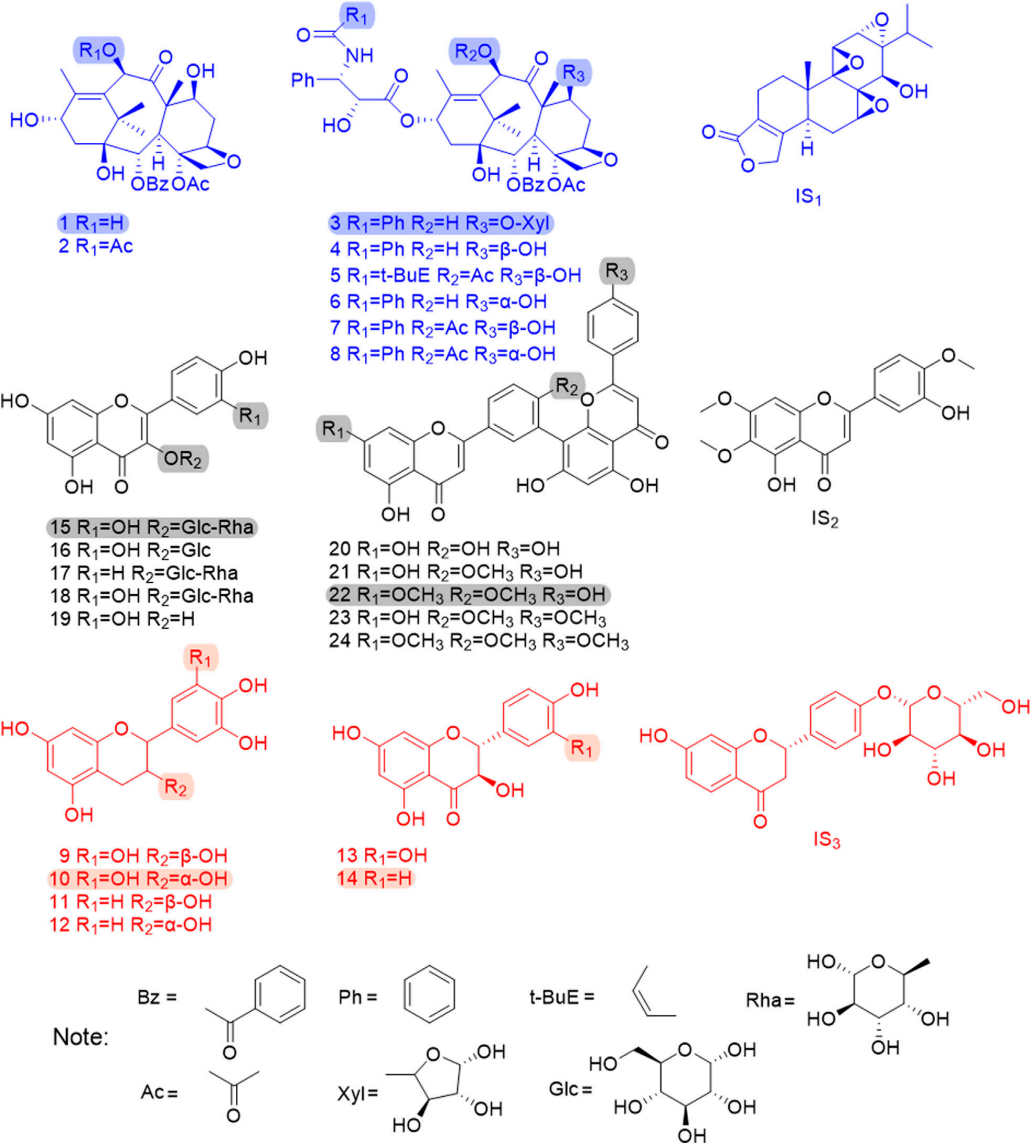
The chemical structures of 24 analytes and 3 internal standards. 1. 10-DAB, 2. BAC, 3. 7-xyl-10-DAT, 4. 10-DAT, 5. CE, 6. 7-epi-10-DAT, 7. TAXOL, 8. 7-epi-TAXOL, 9. GC, 10. EGC, 11. C, 12. EC, 13. TAX, 14. ARO, 15. RT, 16. IQC, 17. NFR, 18. QC, 19. QR, 20. AF, 21. DGK, 22. GK, 23. IGG, 24. SDN, IS1.TP, IS2.CAS, IS3.LIQ; The shadow area shows six chemical quality markers with distinguishing radical pharmacophores.

Methanol, acetonitrile, and formic acid for UPLC analysis were purchased from Merck (Darmstadt, Germany). Deionized water was prepared daily by using a Millipore Milli-Q purification system (Millipore, Bedford, MA, United States). 10-DAB, baccatin III (BAC), 7-xylosyl-10-deacetyltaxol (7-xyl-10-DAT), 10-DAT, cephalomannine (CE), 7-epi-10-deacetyltaxol (7-epi-10-DAT), paclitaxel (TAXOL), 7-epi-paclitaxel (7-epi-TAXOL), gallocatechin (GC), catechin (C), isoquercitrin (IQC), nicotiflorin (NFR), and triptolide (IS_1_) were purchased from Chengdu Mansite Bio-Technology Co., Ltd. (Chengdu, China). Epigallocatechin (EGC), taxifolin (TAX), aromadendrin (ARO), amentoflavone (AF), 7-demethylginkgetin (DGK), GK, isoginkgetin (IGG), and SDN were purchased from Baoji Herbest Bio-Technology Co., Ltd. (Baoji, China). Epicatechin (EC), rutin (RT), QC, quercetin (QR), casticin (IS_2_), and liquiritin (IS_3_) with purities exceeding 98% (determined by HPLC) were purchased from the China National Institute for Food and Drug Control (Beijing, China), and their purity was more than 98% (determined by HPLC). Figure 1 shows the chemical structures of the 24 analytes and the three internal standards.

### 2.3 Preparation of samples and standard solution

Dried *Taxus* leaf samples were ground into 65 mesh powder, weighed accurately to 0.10 g, suspended in 25 mL of 80% methanol aqueous solution, accurately weighed in a 50 mL capped conical flask, and extracted in an ultrasonic bath with an output power of 300 W (40 kHz) for 30 min. The extract was added with 80% methanol to compensate for weight loss. The extract was centrifuged (12,000 r/min, 10 min) then passed through a 0.22 μm microporous membrane. The sample was diluted 5-fold by adding 800 μL of methanol solution to 200 μL of the sample solution. Then, the diluted sample was added to the internal standard mixed solution at a 1:1 *v/v* ratio. All samples were kept at 4°C.

The 24 standards were weighed separately and dissolved in methanol (UPLC–MS grade) to obtain single stock solutions with accurate concentrations. Each stock solution was rediluted and mixed with methanol to obtain a series of working standard solutions, and calibration curves were established by using the mixed working standard solution. All standard solutions were stored in brown glass bottles at 4°C.

Internal standards, namely, triptolide (IS_1_), casticin (IS_2_), and liquiritin (IS_3_), were dissolved in methanol to a concentration of approximately 1 mg/mL individually. Each stock solution was rediluted with methanol to prepare a mixed internal standard solution with 50 ng/mL IS_1_, 50 ng/mL IS_3_, and 100 ng/mL IS_2_. A total of 500 μL of the mixed internal standard was added to 500 μL of the mixed working standard solution or sample solution and filtered through a 0.22 μm micropore membrane before use.

### 2.4 Liquid chromatographic conditions

Chromatographic analysis was performed on a Waters UPLC system (Waters, United States). Twenty-four analytes were chromatographically separated on a Waters Cortecs C_18_ column (2.1 × 100 mm, 1.6 μm) through chromatographic separation. The column temperature was 45°C. The mobile phases consisted of 0.1% formic acid in water (A) and acetonitrile (B). The gradient elution program was as follows: 95% A at 0–0.5 min, 95%–55% A at 0.5–2.5 min, 55% A at 2.5–4.5 min, 55%–25% A at 4.5–8.5 min, 25%–95% A at 8.5–8.6 min, and 95% A at 8.6–10.5 min. The flow rate was 0.2 mL/min, and the volume of the injected sample was 2 μL.

### 2.5 Mass spectrum conditions

Mass spectrometry analysis was conducted with a Waters TQS triple quadrupole mass spectrometer in the switching mode of electrospray positive- and negative-ion modes of multiple reaction monitoring (MRM). The optimized MS conditions were fixed as follows: capillary voltage, 2.50 kV; desolvent gas flow, 800 L/h (N_2_); desolvent gas temperature, 500°C; ion source temperature, 150°C; secondary cone hole extraction voltage, 3.00 V; cone gas flow, 50 L/h (N_2_); collision gas, argon. The most suitable collision energy (COE) and cone voltage (CV) for each analyte was optimized. See Table 2 for the specific parameters.

**TABLE 2:** The retention time (t_R_), precursor ions (MS1), product ions (MS2), CV, COE and ES^+/−^ of the 24 analytes on UPLC-MS/MS.

NO.	Analytes	t_R_ (min)	MS1 (*m*/*z*)	MS2 (*m*/*z*)	CV (V)	COE (eV)	ES^+/−^
1	GC	2.04	304.90	219.06	40	15	ES^−^
2	EGC	2.25	304.90	179.04	30	15	ES^−^
3	C	2.37	289.01	245.01	40	18	ES^−^
4	EC	2.51	289.01	245.01	40	18	ES^−^
5	RT	2.64	608.81	301.03	35	35	ES^-^
6	IQC	2.73	463.20	300.02	40	28	ES^−^
7	NFR	2.75	593.15	285.05	35	30	ES^−^
8	QC	2.88	447.10	301.10	30	23	ES^−^
9	TAX	2.92	303.03	285.03	30	10	ES^−^
10	ARO	3.16	287.05	259.01	30	15	ES^−^
11	QR	3.37	301.08	151.10	30	25	ES^−^
12	10-DAB	3.45	567.20	445.07	40	22	ES^+^
13	AF	3.78	536.90	374.92	40	30	ES^−^
14	BAC	4.03	609.10	549.02	40	22	ES^+^
15	DGK	4.30	551.00	519.06	10	30	ES^−^
16	7-xyl-10-DAT	4.58	966.20	681.10	40	25	ES^+^
17	10-DAT	5.47	834.20	307.94	30	25	ES^+^
18	GK	6.29	565.00	533.15	20	30	ES^−^
19	IGG	6.48	565.00	532.96	10	30	ES^−^
20	CE	6.53	854.20	286.01	40	30	ES^+^
21	7-epi-10-DAT	6.76	834.00	308.01	40	25	ES^+^
22	TAXOL	6.78	876.10	308.08	40	25	ES^+^
23	7-epi-TAXOL	7.66	876.20	308.00	40	30	ES^+^
24	SDN	8.28	578.83	547.10	30	30	ES^−^

### 2.6 Method validation

#### 2.6.1 Calibration curves, LOD, and LOQ

In accordance with the relationship between the peak area ratio of each analyte to IS (*Y*) and the corresponding concentration (*X*) of each analyte in different mixed standard solutions, the established standard curve contained six different concentrations. The slope, intercept, and correlation coefficient (*R* ^2^) of the curve of each analyte were calculated through linear regression analysis method. LOD is an indicators of the sensitivity of methods and instruments. LOQ is the minimum amount of analytes in a sample that can be quantitatively determined. It indicates that analytes with small contents can be accurately quantified through analysis. The LOD and LOQ of each analyte are the concentrations with signal–noise ratios equal to or greater than 3 and 10, respectively, in accordance with the serial dilution of mixed standard solutions.

#### 2.6.2 Precision

The mixed standard solutions with four different concentrations (LOQ, low, middle, and high level) were measured three times a day and re-evaluated for 3 consecutive days. Intra- and interday changes were used as evaluation methods and instrument indicators. The precision changes in the 24 analytes were expressed as the percentage of relative standard deviation (RSD%).

#### 2.6.3 Repeatability and stability

The same six samples of the S3 batch were prepared in accordance with the method described in Section 2.3. The analytes were analyzed by applying this method. The peak area of the 24 analytes was utilized as the evaluation index, and RSD% was used to evaluate repeatability. Moreover, the stability of the samples was studied.

#### 2.6.4 Recovery

The recovery experiment was conducted by adding three standards with different concentrations (50%, 100%, and 150% of the sample) to a known number of samples (S3). The samples were prepared in accordance with the method in Section 2.3 and evaluated on the basis of RSD%.

### 2.7 Data analysis

The quantitative data of the analytes and FDA were statistically analyzed with SPSS statistical software 22.0 (SPSS Inc., Chicago, United States), and the difference was significant. Data were analyzed by Masslynx version 4.2. HCA was performed by using Heatmap Illustrator 1.0. PCA and OPLS-DA were conducted with SIMCA 14.1 software (Umetrics, Umea, Sweden). RF was established with the RF package (version 4.6–14) in the R environment (version 3.5.2).

## 3 Results and discussion

### 3.1 Optimization of chromatographic and mass spectrometry conditions

**FIGURE 2:**
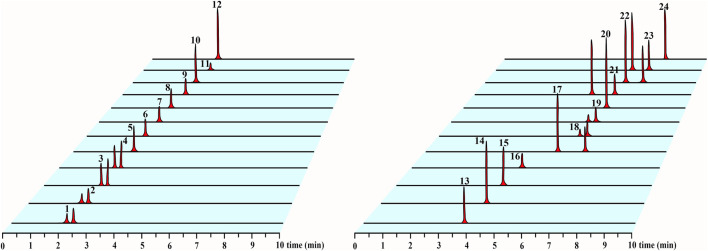
UPLC-MS/MS chromatograms of 24 analytes in mixed standard solution. The sequence of 24 analytes in the figure is consistent with the number in Table 1.

In this experiment, we selected methanol–water, methanol–0.1% formic acid water, acetonitrile–water, and acetonitrile–0.1% formic acid water as the candidate mobile phases and the chromatographic peak separation effect and peak shape as the evaluation indicators. Acetonitrile–0.1% formic acid water was selected on the basis of the optimized result. Our established method separated five isomeric compounds (GC and EGC, C and EC, GK and IGG, 10-DAT and 7-epi-10-DAT, and TAXOL and 7-epi-TAXOL) simultaneously for the first time. To develop a sensitive and accurate quantitative method, we studied the quantitative ion selection of analytes in the positive- and negative-ion ESI modes on the basis of the optimized CV and COE values by using manually optimized MRM parameters, as shown in Table 2. The chromatograms of the representative *T. mairei* sample (S3, methodological validation sample) and mixed standard solution are provided in Supplementary Figure S2 and Figure 2, respectively. Among the 24 analytes measured, we found that four different classes of flavonoids had the best response in the negative-ion mode. Eight taxoids had the best response in the positive-ion mode. IS_1_ was used as the internal standard of taxoids; IS_2_ was utilized as the internal standard of flavanols, flavonols, and biflavones; and IS_3_ was applied as the internal standard of dihydroflavones. ISs could be separated from the chromatographic peaks of the 24 analytes. This characteristic plays an important role in the accurate determination of complex components to ensure the accuracy of results. ISs and the analytes had similar chemical structures, which are indicated by the same color. IS_1_ and taxoids are marked with blue; IS_2_, flavonols, and biflavones are marked with black; and IS_3_, flavanols, and dihydroflavones are marked with red. Refer to Figure 1 for specific analyte results.

### 3.2 Identification of compounds with UPLC–MS/MS

**FIGURE 3:**
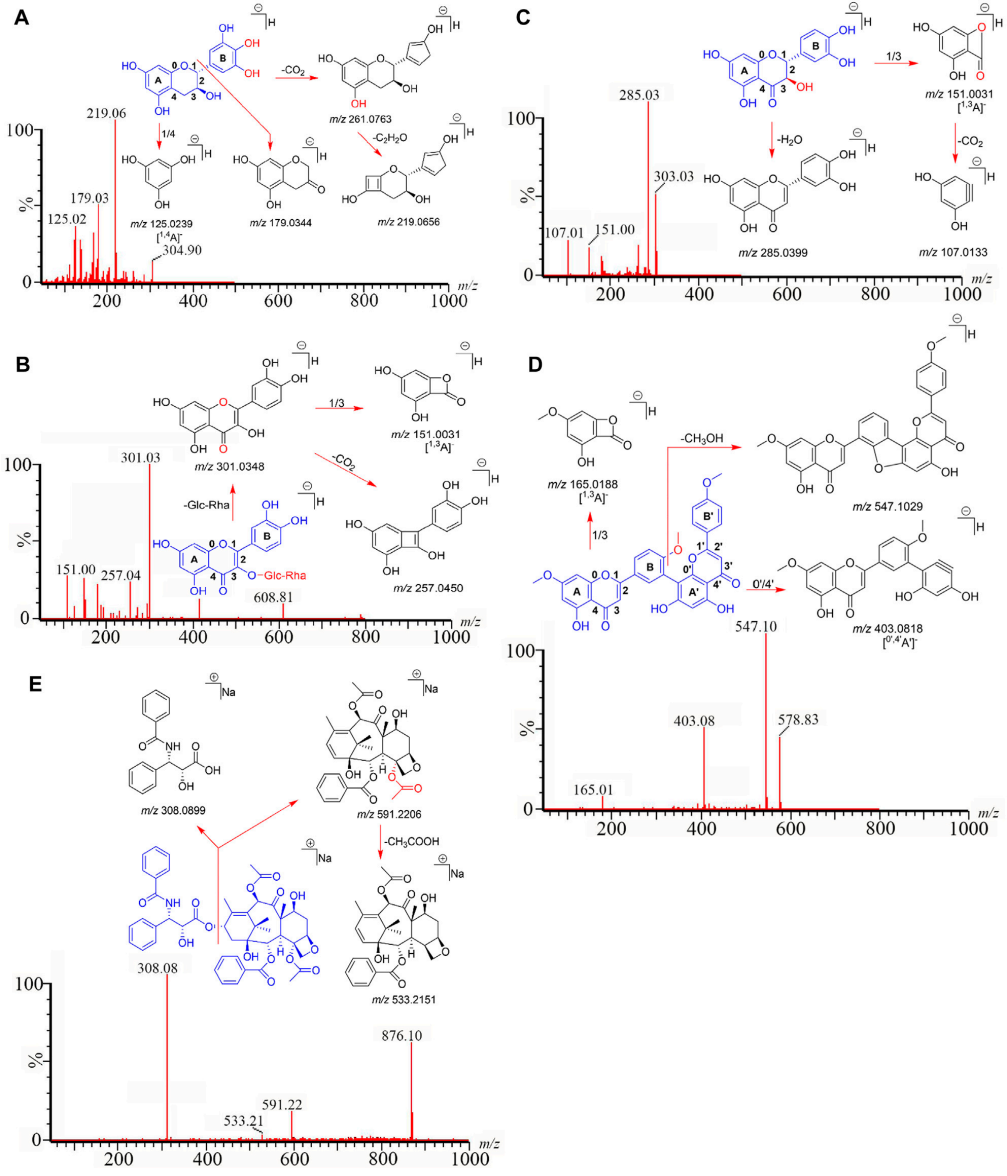
GC **(A)**, RT **(B)**, TAX **(C)**, SDN **(D)**, and TAXOL **(E)** are used as examples to clarify the detailed identification processes of flavanols, flavonols, dihydroflavones, biflavones, and taxoids.

We employed the established analytical method to identify the 24 compounds in six *Taxus* species. Structures were unambiguously assigned on the basis of the retention times and MS spectra of the reference standards (Supplementary Figure S4). The ESI mass spectra provided the characteristic quasi-molecular ions of GC [M–H]^−^ at *m/z* 304.90; EGC [M–H]^−^ at *m/z* 304.90; C [M–H]^−^ at *m/z* 289.01; EC [M–H]^−^ at *m/z* 289.01; RT [M–H]^−^ at *m/z* 608.81; IQC [M–H]^−^ at *m/z* 463.20; NFR [M–H]^−^ at *m/z* 593.15; QC [M–H]^−^ at *m/z* 447.10; QR [M–H]^−^ at *m/z* 301.08; TAX [M–H]^−^ at *m/z* 303.03; ARO [M–H]^−^ at *m/z* 287.05; AF [M–H]^−^ at *m/z* 536.90; DGK [M–H]^−^ at *m/z* 551.00; GK [M–H]^−^ at *m/z* 565.00; IGG [M–H]^−^ at *m/z* 565.00; SDN [M–H]^−^ at *m/z* 578.83; 10-DAB [M–H]^−^ at *m/z* 567.20; BAC [M + Na]^+^ at *m/z* 609.10; 7-xyl-10-DAT [M + Na]^+^ at *m/z* 966.20; 10-DAT [M + Na]^+^ at *m/z* 834.20; CE [M + Na]^+^ at *m/z* 854.20; 7-epi-10-DAT [M + Na]^+^ at *m/z* 834.00; TAXOL [M + Na]^+^ at *m/z* 876.10; and 7-epi-TAXOL [M + Na]^+^ at *m/z* 876.20. Moreover, the characteristic fragmentation behaviors of the 24 compounds revealed by MS/MS analysis in our present study were identical to those in previous studies (Wang Y.-J. et al., 2019; Gai et al., 2020; Luo et al., 2020; Yi et al., 2020) and our previous pre-experimental HPLC–Q-TOF–MS/MS work (Supplementary Figure S3; Supplementary Table S2): *m/z* 305→219→179→125 for GC and EGC; 289→245→203→151→125 for C and EC; *m/z* 609→301→257→151 for RT; *m/z* 463→300→179→151 for IQC; *m/z* 593→285→151 for NFR; *m/z* 447→301→179→151 for QC; *m/z* 303→285→151→107 for TAX, *m/z* 287→259→243→201 for ARO; *m/z* 301→273→179→151→107 for QR; *m/z* 567→531→445 for 10-DAB; *m/z* 537→443→417→399→375 for AF; *m/z* 609→549→427→367 for BAC; *m/z* 551→519→475→457→389 for DGK; *m/z* 966→681→308 for 7-xyl-10-DAT; *m/z* 834→549→308 for 10-DAT and 7-epi-10-DAT; *m/z* 565→533→389→374 for GK and IGG, *m/z* 854→591→286 for CE; *m/z* 876→591→533→308 for TAXAL and 7-epi-TAXOL; *m/z* 579→547→403→165 for SDN, and GC, RT, TAX, SDN, and TAXOL were used as examples to depict the detailed identification processes of flavanols, flavonols, dihydroflavones, biflavones, and taxoids in Figures 3A–E, respectively.

### 3.3 Method validation

#### 3.3.1 Calibration curves, LOD, and LOQ

The regression equation, *R* ^2^, linear range, LOD, and LOQ data of the 24 analytes are shown in Table 3. The calibration curve presented good linearity, and the concentration ranges of the 24 analytes in the samples were within the linear range of the calibration curve. The correlation coefficient (*R* ^2^ = 0.9999–0.9972) of the linear equation was linear. The LOD and LOQ values of the 24 analytes were within the ranges of 0.47–1.53 and 0.94–3.05 ng/mL, respectively.

**TABLE 3:** The linear regression data, LOD and LOQ of the 24 analytes on UPLC-MS/MS.

NO.	Analytes	Regression equation	*R* ^2^	Linear range (ng/mL)	LOD (ng/mL)	LOQ (ng/mL)
1	GC	*Y* = 0.0004*X* + 0.0007	0.9993	2.5625–1,025.0	1.28	2.56
2	EGC	*Y* = 0.0004*X* − 0.0023	0.9972	3.0525–1,221.0	1.53	3.05
3	C	*Y* = 0.0013*X* + 0.0620	0.9990	24.500–9,800.0	1.23	2.45
4	EC	*Y* = 0.0014*X* + 0.0603	0.9992	9.6000–3,840.0	0.48	0.96
5	RT	*Y* = 0.0094*X* + 0.0378	0.9991	5.2750–2,110.0	0.53	1.06
6	IQC	*Y* = 0.0126*X* + 0.0017	0.9997	2.4625–985.00	1.23	2.46
7	NFR	*Y* = 0.0129*X* + 0.0150	0.9993	2.4625–985.00	1.23	2.46
8	QC	*Y* = 0.0138*X* − 0.0241	0.9996	2.7250–1,090.0	1.36	2.73
9	TAX	*Y* = 0.0086*X* + 0.0324	0.9979	1.1640–465.60	0.58	1.16
10	ARO	*Y* = 0.0205*X* + 0.1346	0.9990	0.9760–390.40	0.49	0.98
11	QR	*Y* = 0.0055*X* − 0.0009	0.9997	1.0450–418.00	0.52	1.05
12	10-DAB	*Y* = 0.0033*X* + 0.1629	0.9981	1.0480–4,192.0	0.52	1.05
13	AF	*Y* = 0.0825*X* − 0.0368	0.9983	0.9400–376.00	0.47	0.94
14	BAC	*Y* = 0.0729*X* + 0.3102	0.9994	1.1200–448.00	0.56	1.12
15	DGK	*Y* = 0.0974*X* − 0.0167	0.9979	1.0080–403.20	0.50	1.01
16	7-xyl-10-DAT	*Y* = 0.0090*X* + 0.0035	0.9996	1.1080–443.20	0.55	1.11
17	10-DAT	*Y* = 0.0441*X* + 0.1235	0.9996	1.1720–468.80	0.59	1.17
18	GK	*Y* = 0.0191*X* − 0.0066	0.9998	2.6400–1,056.0	1.32	2.64
19	IGG	*Y* = 0.0378*X* − 0.0029	0.9997	1.9440–777.60	0.97	1.94
20	CE	*Y* = 0.0787*X* − 0.0500	0.9996	1.1440–457.60	0.57	1.14
21	7-epi-10-DAT	*Y* = 0.0275*X* + 0.0009	0.9992	0.9800–392.00	0.49	0.98
22	TAXOL	*Y* = 0.0383*X* + 0.0295	0.9999	1.9920–796.80	1.00	1.99
23	7-epi-TAXOL	*Y* = 0.0277*X* − 0.0005	0.9997	1.1880–475.20	0.59	1.19
24	SDN	*Y* = 0.0098*X* + 0.7310	0.9985	10.520–4,208.0	0.53	1.05

#### 3.3.2 Precision, repeatability, and stability

The intra- and interday RSD% values of the peak areas of the 24 analytes were less than 6.72% (*n* = 3) and 6.83% (*n* = 3) respectively, as presented in Table 4. The RSD% values of repeatability and stability were 1.93%–7.58% (*n* = 6) and 1.98%–5.30% (*n* = 6), respectively, as shown in Table 5. The results show that the proposed method and instrument in our present work have better accuracy than those in previous studies.

**TABLE 4:** Precision and accuracy for the 24 analytes.

NO.	Analytes	Concentration (ng/mL)	Precision (*n* = 3, RSD/%)		NO.	Analytes	Concentration (ng/mL)	Precision (*n* = 3, RSD/%)	
			Intra-day	Inter-day				Intra-day	Inter-day
1	GC	2.56	5.28	4.84	13	AF	0.94	5.90	4.64
		10.25	5.06	4.93			3.76	5.32	4.08
		102.50	4.43	5.17			37.60	3.85	5.02
		1,025.00	4.88	5.02			376.00	3.51	4.96
2	EGC	3.05	6.23	4.85	14	BAC	1.12	4.67	4.51
		12.21	3.24	3.86			4.48	3.92	4.75
		122.10	4.82	4.77			44.80	4.36	3.50
		1,221.00	4.34	5.32			448.00	2.81	5.34
3	C	2.45	5.16	4.82	15	DGK	1.01	4.97	5.77
		98.00	4.28	4.03			4.03	3.64	4.10
		980.00	5.34	4.36			40.32	3.66	4.83
		9,800.00	5.81	5.48			403.20	5.92	4.84
4	EC	0.96	4.87	5.31	16	7-xyl-10-DAT	1.11	5.02	5.16
		38.40	4.35	4.96			4.43	4.23	4.51
		384.00	2.83	3.82			44.32	1.60	5.51
		3,840.00	4.64	4.49			443.20	4.93	5.04
5	RT	1.06	3.78	4.36	17	10-DAT	1.17	6.72	6.18
		21.10	4.61	4.57			4.69	4.33	4.97
		211.00	2.74	5.39			46.88	2.71	4.46
		2,110.00	3.82	3.91			468.80	4.35	5.52
6	IQC	2.46	6.65	4.55	18	GK	2.64	5.69	4.91
		9.85	3.87	3.50			10.56	3.20	4.03
		98.50	1.32	3.26			105.60	5.48	5.02
		985.00	5.03	4.54			1,056.00	5.43	4.11
7	NFR	2.46	4.98	5.72	19	IGG	1.94	4.25	5.27
		9.85	4.33	4.54			7.78	1.92	3.75
		98.50	4.26	4.71			77.76	4.78	4.13
		985.00	4.12	3.16			777.60	3.65	3.12
8	QC	2.73	3.32	4.53	20	CE	1.14	5.63	6.01
		10.90	4.88	5.86			4.58	4.72	4.94
		109.00	3.92	5.31			45.76	1.53	3.68
		1,090.00	5.33	4.35			457.60	5.05	3.59
9	TAX	1.16	4.90	5.03	21	7-epi-10-DAT	0.98	5.58	5.72
		4.66	4.26	5.01			3.92	5.26	4.25
		46.56	5.21	4.54			39.20	4.71	4.04
		465.60	5.42	5.63			392.00	3.94	4.25
10	ARO	0.98	5.39	4.72	22	TAXOL	1.99	4.82	5.44
		3.90	3.21	5.31			7.97	3.71	5.28
		39.04	1.92	3.49			79.68	4.52	4.69
		390.40	1.95	3.98			796.80	3.14	4.32
11	QR	1.05	1.84	6.83	23	7-epi-TAXOL	1.19	5.15	4.78
		4.18	6.34	5.72			4.75	4.62	4.45
		41.80	4.92	5.47			47.52	4.06	4.73
		418.00	4.58	3.46			475.20	5.13	4.52
12	10-DAB	1.05	5.63	5.31	24	SDN	1.05	1.96	2.63
		41.92	4.32	4.26			42.08	3.69	5.31
		419.20	3.03	3.51			420.80	5.25	4.27
		4,192.00	4.93	5.77			4,208.00	4.92	3.84

**TABLE 5:** Stability and repeatability for the 24 analytes in the leaves of *Taxus* species.

NO.	Analytes	Stability (*n* = 6, RSD/%)	Repeatability (*n* = 6, RSD/%)
1	GC	3.86	1.93
2	EGC	4.38	3.76
3	C	3.43	5.88
4	EC	3.14	6.57
5	RT	4.49	5.06
6	IQC	3.50	2.34
7	NFR	4.10	3.06
8	QC	3.82	7.01
9	TAX	2.02	5.78
10	ARO	2.65	4.20
11	QR	4.78	5.78
12	10-DAB	3.24	2.04
13	AF	4.96	6.12
14	BAC	3.90	5.34
15	DGK	1.98	2.33
16	7-xyl-10-DAT	3.58	5.13
17	10-DAT	3.69	7.36
18	GK	4.91	6.32
19	IGG	5.30	7.44
20	CE	3.13	5.17
21	7-epi-10-DAT	2.70	4.36
22	TAXOL	3.09	6.95
23	7-epi-TAXOL	4.82	4.02
24	SDN	4.50	7.58

#### 3.3.3 Recovery

As shown in Table 6, the average recoveries of the 24 analytes at three different concentration levels ranged from 89.11% ± 4.37%–105.29% ± 2.78% with the RSD% of 1.03%–9.92% (*n* = 3). These results demonstrate that our method has good recovery.

**TABLE 6:** Recovery for the 24 analytes.

NO.	Analytes	Content of compounds in the sample (μg)	Spiked content of compounds (μg)	Measured content of compounds (μg)	Averagerecovery (%, *n* = 3)	RSD (%, *n* = 3)	NO.	Analytes	Content of compounds in the sample (μg)	Spiked content of compounds (μg)	Measured content of compounds (μg)	Averagerecovery (%, *n* = 3)	RSD (%, *n* = 3)
1	GC	30.18 ± 0.40	16.40	46.20 ± 0.61	97.66 ± 1.29	1.32	13	AF	6.10 ± 0.06	2.82	9.07 ± 0.13	105.29 ± 2.78	2.64
		30.36 ± 0.75	30.75	60.55 ± 0.56	98.16 ± 1.89	1.92			6.14 ± 0.08	6.11	12.19 ± 0.19	99.00 ± 1.99	2.01
		30.44 ± 0.41	45.10	76.21 ± 1.07	99.31 ± 3.47	3.50			6.17 ± 0.07	9.40	14.85 ± 0.45	92.39 ± 4.07	4.41
2	EGC	63.02 ± 0.85	30.53	92.20 ± 1.40	95.58 ± 1.94	2.03	14	BAC	1.22 ± 0.02	0.62	1.79 ± 0.03	92.29 ± 2.78	3.02
		63.55 ± 0.74	61.05	123.88 ± 1.01	98.83 ± 1.12	1.13			1.23 ± 0.02	1.23	2.36 ± 0.04	91.64 ± 2.13	2.33
		63.68 ± 0.89	91.58	154.21 ± 1.81	98.85 ± 1.02	1.03			1.24 ± 0.02	1.85	3.08 ± 0.04	99.29 ± 1.21	1.22
3	C	127.66 ± 1.14	68.60	194.97 ± 3.01	98.12 ± 2.79	2.84	15	DGK	4.22 ± 0.01	2.52	6.82 ± 0.11	102.88 ± 3.98	3.87
		127.70 ± 0.82	127.40	249.50 ± 4.51	95.61 ± 2.96	3.09			4.24 ± 0.04	3.78	7.94 ± 0.19	97.87 ± 4.09	4.18
		128.40 ± 0.88	196.00	319.72 ± 3.04	97.61 ± 1.11	1.14			4.26 ± 0.04	6.30	10.48 ± 0.29	98.78 ± 3.97	4.02
4	EC	31.90 ± 0.81	15.36	46.11 ± 0.79	92.56 ± 5.70	6.16	16	7-xyl-10-DAT	13.82 ± 0.07	6.65	20.29 ± 0.24	97.26 ± 2.86	2.94
		32.28 ± 0.54	33.60	65.01 ± 1.45	97.42 ± 2.70	2.77			13.84 ± 0.03	13.30	26.69 ± 0.48	96.66 ± 3.55	3.67
		32.64 ± 0.44	48.00	81.26 ± 1.23	101.28 ± 2.77	2.73			13.87 ± 0.12	20.50	32.32 ± 1.08	90.03 ± 4.66	5.18
5	RT	241.03 ± 5.85	126.60	369.06 ± 14.05	101.13 ± 6.61	6.53	17	10-DAT	1.90 ± 0.03	1.17	3.04 ± 0.04	97.18 ± 3.57	3.68
		242.63 ± 4.04	242.65	482.31 ± 7.33	98.78 ± 2.08	2.11			1.91 ± 0.04	1.76	3.71 ± 0.07	102.13 ± 1.99	1.95
		245.75 ± 1.12	369.25	611.02 ± 5.71	98.92 ± 1.25	1.27			1.94 ± 0.03	2.93	4.75 ± 0.12	96.02 ± 3.52	3.67
6	IQC	19.26 ± 0.13	9.85	28.76 ± 0.30	96.42 ± 3.20	3.32	18	GK	38.89 ± 0.24	19.80	59.53 ± 2.21	104.22 ± 10.06	9.65
		19.33 ± 0.19	19.7	39.61 ± 1.56	102.93 ± 6.96	6.76			38.95 ± 0.13	39.60	79.32 ± 1.40	101.93 ± 3.26	3.20
		19.40 ± 0.24	29.55	49.11 ± 0.87	100.54 ± 2.43	2.42			39.11 ± 0.15	58.08	97.07 ± 1.49	99.79 ± 2.32	2.33
7	NFR	35.93 ± 0.33	17.73	53.26 ± 0.59	97.75 ± 1.52	1.56	19	IGG	1.28 ± 0.02	0.68	1.90 ± 0.04	90.68 ± 3.95	4.36
		35.97 ± 0.30	35.46	69.67 ± 0.47	95.02 ± 1.59	1.68			1.29 ± 0.01	1.26	2.58 ± 0.05	101.86 ± 3.63	3.56
		36.21 ± 0.24	54.18	89.81 ± 1.81	98.94 ± 3.14	3.17			1.30 ± 0.02	1.94	3.22 ± 0.07	99.01 ± 2.67	2.70
8	QC	6.35 ± 0.11	3.27	9.29 ± 0.32	89.90 ± 6.60	7.34	20	CE	2.70 ± 0.03	1.43	4.09 ± 0.08	96.99 ± 3.29	3.40
		6.40 ± 0.13	6.54	12.75 ± 0.26	97.20 ± 2.10	2.17			2.71 ± 0.04	2.86	5.40 ± 0.29	94.09 ± 8.77	9.32
		6.44 ± 0.12	9.81	15.54 ± 0.42	92.77 ± 3.22	3.48			2.73 ± 0.03	4.29	7.00 ± 0.23	99.35 ± 4.51	4.54
9	TAX	0.90 ± 0.01	0.47	1.37 ± 0.04	99.96 ± 6.41	6.41	21	7-epi-10-DAT	2.34 ± 0.03	1.23	3.49 ± 0.06	93.66 ± 2.82	3.01
		0.90 ± 0.01	0.87	1.68 ± 0.04	89.11 ± 4.37	4.90			2.35 ± 0.02	2.45	4.74 ± 0.21	97.60 ± 7.61	7.80
		0.91 ± 0.01	1.40	2.26 ± 0.07	96.46 ± 4.67	4.84			2.37 ± 0.02	3.68	6.11 ± 0.12	101.82 ± 2.54	2.50
10	ARO	1.58 ± 0.02	0.78	2.34 ± 0.07	97.56 ± 6.70	6.87	22	TAXOL	2.10 ± 0.03	1.49	3.54 ± 0.06	96.39 ± 2.73	2.83
		1.61 ± 0.04	1.56	3.11 ± 0.05	96.12 ± 1.09	1.13			2.11 ± 0.03	2.99	4.98 ± 0.22	96.06 ± 6.44	6.70
		1.63 ± 0.04	2.34	3.88 ± 0.06	95.93 ± 2.95	3.08			2.13 ± 0.02	3.98	6.12 ± 0.06	100.08 ± 1.26	1.26
11	QR	0.29 ± 0.00	0.13	0.42 ± 0.01	99.33 ± 4.61	4.64	23	7-epi-TAXOL	0.33 ± 0.00	0.18	0.50 ± 0.02	91.87 ± 7.97	8.67
		0.29 ± 0.00	0.25	0.55 ± 0.01	101.14 ± 1.35	1.34			0.33 ± 0.00	0.36	0.66 ± 0.03	91.45 ± 7.03	7.68
		0.30 ± 0.01	0.38	0.67 ± 0.01	99.60 ± 1.03	1.03			0.34 ± 0.00	0.48	0.81 ± 0.04	99.90 ± 7.05	7.06
12	10-DAB	2.84 ± 0.03	1.57	4.37 ± 0.07	97.26 ± 2.29	2.35	24	SDN	198.46 ± 3.20	99.94	296.93 ± 12.94	98.52 ± 9.78	9.92
		2.85 ± 0.02	2.62	5.35 ± 0.06	95.30 ± 2.25	2.36			199.13 ± 4.34	199.88	392.10 ± 11.79	96.54 ± 3.92	4.06
		2.88 ± 0.02	4.19	7.05 ± 0.10	99.56 ± 1.76	1.77			201.46 ± 2.04	299.82	503.54 ± 8.58	100.75 ± 2.20	2.19

The 24 analytes presented a good linear relationship in the tested concentration range, and their detection limits, precision, accuracy, stability, repeatability, and recovery met the requirements.

### 3.4 Quantification of the 24 analytes in the leaves of *Taxus* species samples

**FIGURE 4:**
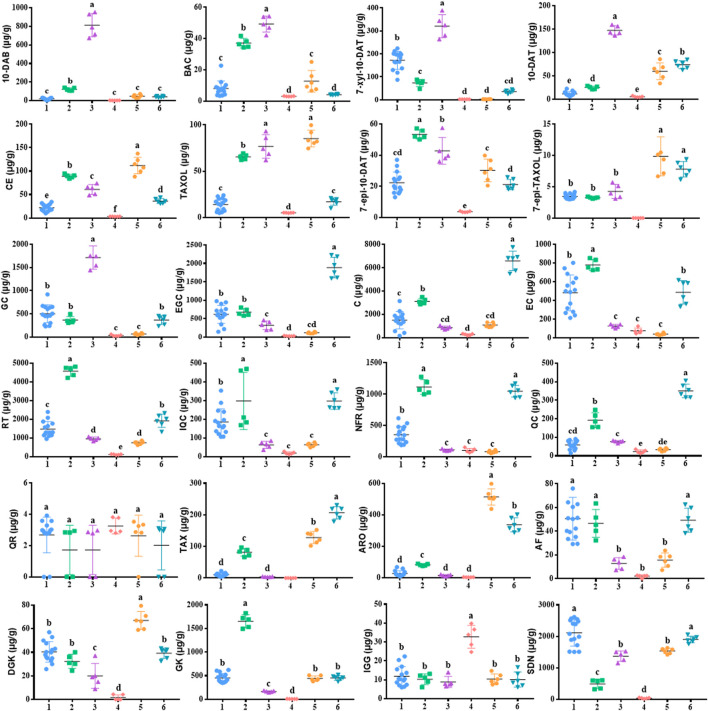
Comparative analysis of 24 components in 42 leaf samples from the six *Taxus* species. The mean ± SDs not sharing the same lowercase letters are signifificantly different (*p* < 0.05). 1: *T. mairei*, 2: *T. chinensis*, 3: *T. yunnanensis*, 4: T. *wallichiana*, 5: *T. cuspidata*, 6: *T. media*.

Eight taxoids, four flavanols, five flavonols, two dihydroflavones, and five biflavones in 42 leaf samples from *Taxus* species were determined and analyzed by UPLC–MS/MS after method validation (See Figure 4; 1: *T. mairei*, 2: *T. chinensis*, 3: *T. yunnanensis*, 4: *T. wallichiana*, 5: *T. cuspidata*, 6: *T. media*). Under the UPLC–MS/MS condition, all analytes can be detected in the leaves of the different *Taxus* species. The contents of chemical components in different *Taxus* species were obviously different. We first discuss toxoids. In *T. wallichiana*, the amount of 7-Epi-TAXOL was less than the LOQ. Among the eight taxoids, 10-DAB, BAC, 7-xyl-10-DAT, 10-DAT, and TAXOL were higher in *T. yunnanensis* than in the other five *Taxus* species; CE, TAXOL, and 7-epi-TAXOL were higher in *T. cuspidata* than in the other five *Taxus* species; and 7-epi-10-DAT was higher in *T. chinensis* than in the other five *Taxus* species. The eight other taxoids were abundant in *T. yunnanensis* and had low abundance in *T. wallichiana* (*T. yunnanensis > T. cuspidate > T. chinensis > T. media > T. mairei > T. wallichiana*). Therefore, *T. yunnanensis* was used as a plant material for taxoid extraction. Second, we discuss flavanols. We found that among the four flavanols, GC was higher in *T. yunnanensis* than in the other *Taxus* species, and EGC, C, and EC were higher in *T. mairei*, *T. chinensis*, and *T. media* than in the other three *Taxus* species. Third, we found that among the five flavonols, QR was present at low levels in the six *Taxus* species. RT, IQC, NFR, and QC were higher in *T. maireis*, *T. chinensis*, and *T. media* than in the other *Taxus* species. Fourth, we discovered that of the two dihydroflavones, TAX and ARO were higher in *T. cuspidata* and *T. media* than in the other *Taxus* species. Fifth, among the five biflavones, AF was higher in *T. mairei*, *T. chinensis*, and *T. media* than in the other *Taxus* species. DGK was higher in *T. cuspidata*, GK was higher in *T. chinensis*, IGG was higher in *T. wallichiana*, and SDN was higher in *T. mairei* than in the other *Taxus* species*.* The components in the leaves of different *Taxus* species were different, and quantitative analysis could aid the effective use of the leaves of different *Taxus* varieties. Moreover, we comparatively analyzed the quantitative results of 24 analytes from the six *Taxus* species on the basis of their mean ± SDs in Figure 4 **,** and the content determination results are provided in Table 7.

**TABLE 7:** Content results of 24 analytes.

NO.	Content of analytes (μg/g)											
	GC	EGC	C	EC	RT	IQC	NFR	QC	TAX	ARO	QR	10-DAB
S1	650.42	340.78	553.82	242.83	2080.78	246.08	617.16	74.31	7.53	36.08	2.97	10.69
S2	453.40	610.88	411.06	215.66	1,637.05	156.85	188.33	33.29	9.44	17.64	2.81	4.28
S3	304.64	630.77	1,273.56	318.37	2,394.99	191.08	357.87	63.32	8.99	15.74	2.93	28.37
S4	230.17	590.93	1,182.50	281.55	1768.06	181.25	283.39	55.28	5.78	17.54	2.99	18.14
S5	258.65	714.30	158.92	268.99	948.54	121.58	281.00	29.39	5.94	10.90	2.80	32.20
S6	239.34	620.76	1,698.03	561.52	1,137.64	132.12	538.14	53.32	13.04	19.52	3.58	12.55
S7	461.06	758.40	1,347.46	501.26	1,292.47	136.14	236.23	81.31	12.05	58.05	—	6.15
S8	486.28	716.49	1,453.23	513.76	1,238.21	240.29	193.33	91.66	9.55	67.46	3.92	13.42
S9	639.40	943.69	1882.09	592.15	1878.95	288.06	441.84	51.28	5.28	10.30	3.29	31.28
S10	671.84	521.30	1,649.97	369.85	1,294.88	106.69	217.14	15.29	10.06	24.87	3.03	4.42
S11	652.81	214.91	3,146.21	746.37	1,153.79	353.35	346.31	86.29	22.41	23.40	2.76	26.21
S12	451.13	143.04	1799.07	492.25	1,110.31	196.53	275.13	56.31	3.88	16.18	3.59	19.05
S13	920.28	546.86	1,636.01	641.41	1,567.02	163.60	479.48	81.15	16.30	43.76	2.78	22.23
S14	506.31	903.44	2,276.12	686.68	1,197.46	163.62	403.35	40.29	13.91	16.30	—	29.80
S15	606.07	974.48	2008.58	804.85	1,301.66	106.23	478.78	72.28	11.58	12.79	2.79	22.62
S16	349.42	801.66	3,240.97	755.30	4,367.48	469.80	1,072.79	217.67	71.87	74.68	—	108.87
S17	322.74	632.74	2,861.41	835.99	4,698.35	168.48	1,192.78	186.11	66.27	78.15	2.84	119.83
S18	356.83	602.44	3,077.55	854.99	4,757.14	183.70	1,277.08	156.12	96.78	89.19	—	108.26
S19	307.16	578.57	3,486.67	725.99	4,218.94	209.27	1,027.66	154.96	88.77	79.90	2.94	137.68
S20	496.04	741.70	2,957.88	734.35	4,813.86	460.90	999.35	249.06	83.70	86.40	2.85	140.69
S21	1,545.84	327.76	709.82	103.58	893.48	35.98	95.68	82.92	3.13	12.91	—	954.70
S22	2095.95	455.94	1,050.02	122.43	991.28	84.67	103.28	76.25	4.60	7.42	—	675.19
S23	1,441.02	206.00	843.26	145.38	828.96	66.43	130.35	78.28	3.37	20.87	2.97	791.40
S24	1743.72	403.08	912.65	147.30	977.00	74.62	120.61	80.37	4.76	18.31	2.79	715.97
S25	1754.28	191.28	781.88	120.08	1,065.56	53.30	105.19	67.15	3.99	12.66	2.87	930.83
S26	22.02	29.19	334.35	47.73	121.37	10.51	68.08	12.94	—	2.63	2.91	2.85
S27	40.84	24.86	281.95	72.45	132.41	14.75	86.69	18.91	—	3.14	3.82	3.46
S28	58.43	37.56	190.15	75.98	93.32	21.97	120.30	28.60	—	2.52	3.78	3.65
S29	23.60	28.32	173.99	127.41	80.79	27.74	152.50	36.20	—	4.31	2.81	3.12
S30	21.92	26.39	313.67	61.50	123.97	15.63	89.53	20.24	—	2.57	2.95	2.84
S31	61.59	125.68	909.28	41.95	665.99	64.53	81.63	34.06	112.82	505.20	3.53	45.09
S32	68.11	121.88	1,097.12	43.08	755.06	69.66	78.94	36.81	138.83	532.86	3.34	54.39
S33	40.28	144.46	898.65	27.83	613.83	48.92	99.63	22.20	102.08	501.71	2.87	30.71
S34	51.70	106.31	975.03	34.43	863.22	54.78	110.16	26.09	121.03	437.00	—	27.53
S35	85.58	95.38	1,321.15	58.88	766.83	76.93	69.98	42.46	152.68	597.05	3.25	73.82
S36	88.47	89.67	1,310.73	39.79	763.43	65.66	54.12	38.80	140.97	509.63	2.83	61.22
S37	271.68	1,614.24	5,763.44	435.80	1,325.62	260.65	1,134.73	406.58	230.41	314.90	3.00	42.41
S38	234.46	1754.07	5,489.43	364.77	2,187.18	301.90	951.74	333.62	202.37	379.58	3.07	51.08
S39	438.38	1,592.00	6,819.06	334.76	2,323.43	359.38	1,059.37	316.03	190.33	401.05	3.11	39.79
S40	376.72	2,184.79	6,925.25	618.89	1897.49	262.92	941.53	355.26	218.99	301.85	2.93	31.74
S41	453.91	1953.29	6,827.16	587.11	1752.84	259.20	1,031.42	379.08	178.81	287.49	—	34.77
S42	421.08	2,225.94	7,765.34	585.67	2018.46	342.20	1,170.43	315.42	218.82	334.02	—	49.47

NO.	Content of analytes (μg/g)											
	AF	BAC	DGK	7-xyl-10-DAT	10-DAT	GK	IGG	CE	7-epi-10-DAT	TAXOL	7- epi-TAXOL	SDN
S1	40.31	6.27	57.10	129.76	8.33	483.50	6.03	16.71	25.79	5.06	3.08	2,391.31
S2	50.37	8.64	30.01	87.40	10.49	361.64	8.65	14.31	19.21	6.04	3.38	1920.19
S3	60.47	12.19	42.06	137.66	18.85	387.67	12.83	27.06	23.48	21.06	3.31	1988.01
S4	64.36	4.21	38.04	163.97	13.16	365.19	9.69	24.47	37.06	17.23	4.18	1892.02
S5	51.29	22.68	35.03	183.50	10.10	415.54	7.40	28.10	16.52	16.27	3.09	2,613.88
S6	92.34	3.75	35.01	194.35	15.48	397.66	10.33	29.56	20.18	23.03	3.44	2,510.78
S7	39.37	7.87	50.00	165.42	10.20	587.18	22.41	17.22	18.89	8.52	3.05	2,477.10
S8	29.30	8.35	40.05	202.51	7.05	608.25	10.59	19.55	15.86	13.74	3.68	2,571.57
S9	76.06	8.95	47.62	223.85	22.94	624.06	20.47	10.08	13.09	9.26	3.97	2,404.03
S10	51.03	3.58	25.82	165.54	6.66	348.96	7.06	13.25	15.47	5.78	3.01	1,516.19
S11	62.84	10.28	52.97	201.28	11.12	520.20	13.70	19.56	24.73	24.12	3.85	1,508.35
S12	42.31	3.32	36.08	212.30	13.60	468.94	6.36	31.71	29.53	7.34	3.11	2,547.22
S13	33.59	7.29	43.04	118.34	9.03	511.25	15.55	25.09	17.88	14.94	3.99	1,511.48
S14	29.40	5.91	33.00	197.20	9.97	359.25	17.04	34.39	33.20	18.72	3.62	2081.35
S15	35.26	7.62	40.27	197.12	15.01	375.70	10.48	17.94	24.85	20.44	3.06	1720.56
S16	40.38	41.72	36.09	81.74	26.54	1,493.41	11.10	86.81	57.26	62.30	3.29	345.43
S17	32.31	38.17	31.02	73.78	21.65	1,691.65	13.16	90.81	52.01	63.04	3.09	599.64
S18	51.58	34.38	24.40	76.36	22.14	1721.91	12.23	90.36	50.20	65.85	3.27	581.54
S19	45.40	34.78	40.09	48.60	29.06	1823.46	9.07	94.78	51.77	69.44	3.19	606.92
S20	63.42	36.35	30.15	88.07	27.38	1,531.70	6.07	83.80	55.88	67.29	3.43	321.85
S21	16.37	54.23	20.04	263.65	136.90	147.26	13.97	66.07	57.58	93.22	5.70	1,525.29
S22	8.39	46.95	9.00	388.89	156.40	159.13	7.68	51.27	42.70	68.84	3.11	1,149.18
S23	18.50	42.12	19.02	324.59	159.99	187.18	6.57	74.03	35.95	74.27	5.12	1,427.23
S24	7.36	49.11	15.04	343.91	143.24	176.94	7.88	48.82	38.12	62.12	3.38	1,253.06
S25	13.84	53.92	37.13	280.34	142.26	167.32	8.25	63.98	39.76	86.03	3.94	1,507.01
S26	2.45	3.19	4.34	2.84	7.91	13.02	39.82	3.40	3.34	5.48	—	56.24
S27	2.89	3.20	—	2.85	6.04	7.93	28.64	3.39	4.05	5.33	—	49.83
S28	—	2.96	—	2.86	4.65	8.68	33.84	3.03	3.47	5.10	—	30.53
S29	2.54	2.96	4.21	2.98	4.80	8.74	36.65	3.35	4.16	5.37	—	28.35
S30	2.52	2.94	—	3.13	5.61	8.65	24.74	3.16	3.49	5.38	—	44.50
S31	18.59	15.96	59.78	2.95	58.94	507.90	7.54	107.63	20.53	92.05	6.67	1,460.95
S32	14.77	9.22	66.29	2.88	65.85	425.58	10.30	137.55	39.83	80.51	10.49	1,526.92
S33	7.39	11.73	59.04	3.23	47.84	509.02	12.34	99.39	31.28	99.82	15.39	1,421.22
S34	10.80	25.07	67.06	2.85	34.42	421.20	14.74	88.25	28.86	83.27	7.16	1,632.79
S35	23.82	7.58	79.53	3.09	86.01	409.98	8.10	123.56	24.33	74.64	10.14	1,594.54
S36	18.93	6.64	70.88	3.41	68.86	381.08	9.23	115.00	36.67	82.20	9.38	1,583.31
S37	50.88	3.92	43.39	34.73	67.53	431.47	7.05	40.62	20.56	19.90	6.14	1846.89
S38	43.86	4.24	41.30	32.12	62.93	375.88	16.65	30.50	24.74	17.82	8.10	2059.95
S39	38.38	4.57	32.49	25.35	77.20	459.19	6.09	35.61	19.32	10.03	8.83	1916.82
S40	59.67	4.04	42.24	45.57	85.16	485.66	8.53	34.86	18.56	21.08	6.85	1759.02
S41	62.44	4.13	35.08	40.59	71.68	458.40	9.40	31.18	18.18	18.86	7.53	1874.85
S42	40.61	3.71	40.85	39.18	81.16	518.46	13.12	43.75	25.84	15.73	9.41	1974.25

### 3.5 Heatmap and HCA

**FIGURE 5:**
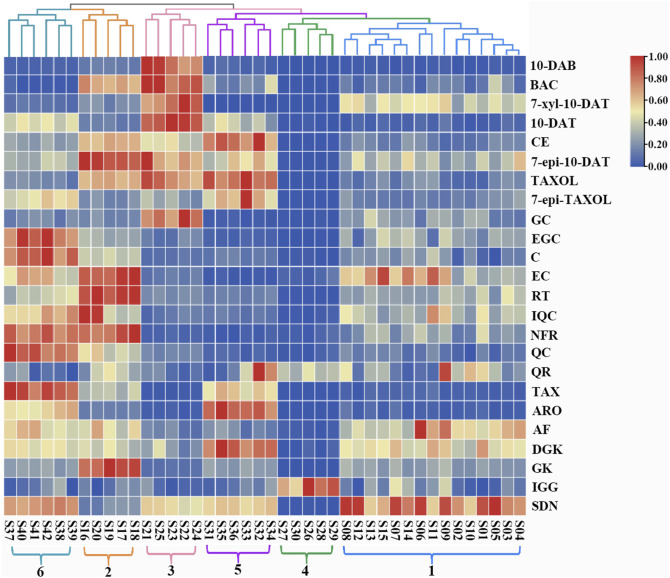
Heatmap and dendrogram of HCA in 42 leaf samples from the six *Taxus* species. S1-S42 represents 42 leaf samples from the six *Taxus* species. 1: *T. mairei*, 2: *T. chinensis*, 3: *T. yunnanensis*, 4: *T. wallichiana*, 5: *T. cuspidata*, 6: *T. media*, and 24 analyte names are shown at the right of the figure. The colors from red, yellow and blue represent the content of analytes in the sample from high to low.

HCA in Heatmap Illustrator 1.0 software was used to analyze the contents of the 24 analytes in the leaves of six *Taxus* species, and the content data were normalized. The clustering method was the Ward method, and the distance type was square Euclidean distance. The HCA results are shown in Figure 5. The 42 batches of leaf samples from the six *Taxus* species divided into six categories. Therefore, the established content determination method can distinguish the six different varieties of *Taxus* species. The differences in the contents of the 24 analytes in the leaves of different *Taxus* species can be clearly seen in Figure 5. Among the eight taxoids, 10-DAB, BAC, 7-xyl-10-DAT, and 10-DAT were higher in *T. yunnanensis*; CE, TAXOL, and 7-epi-TAXOL were higher in *T. cuspidata*; and 7-epi-10-DAT was higher in *T. chinensis* than in the other species. The contents of four flavanols and five flavonols were higher in *T. chinensis* and *T. media* than in the other *Taxus* species. However, GC was higher in *T. yunnanensis* than in the other *Taxus* species. The contents of two dihydroflavones were higher in *T. cuspidata* and *T. media* than in the other *Taxus* species. Among the biflavones, DGK was higher in *T. cuspidata* than in the other species*.* GK content was higher in *T. chinensis*, IGG content was higher in *T. wallichiana*, and SDN content was higher in *T. mairei* than in the other *Taxus* species. HCA revealed the differences in the contents of the 24 analytes in leaf samples from six different *Taxus* species with high intuitiveness.

### 3.6 PCA and OPLS-DA

**FIGURE 6:**
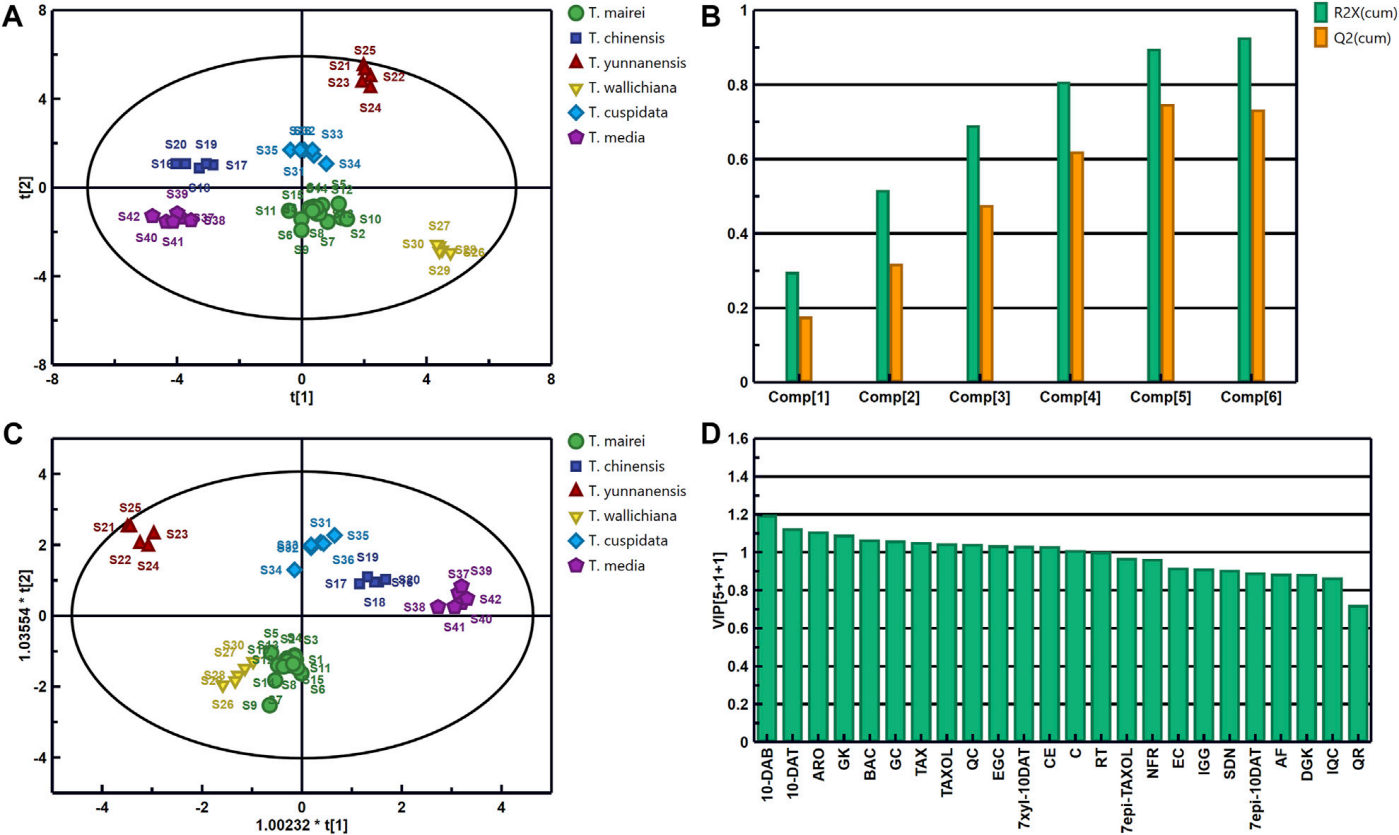
Scores **(A)** and Accumulated R^2^X and Q^2^ with different numbers of principal components **(B)** of PCA; scores **(C)** of OPLS-DA and the variable importance predictive value (VIP) of 24 analytes **(D)** in 42 leaf samples from the six *Taxus* species.

PCA is the most commonly data analysis method in the statistical analysis of multiple elements. In this method, the information of original features is retained to the maximum extent without the loss of important information (Jolliffe and Cadima, 2016; Giuliani, 2017). We imported the data into Simca 14.1 with sample batch as the observation value and the 24 analytes as the variables. Unit variance scaling was used for data normalization to generate the PCA model. The scores and accumulated R^2^X and Q^2^ with different numbers of principal components are displayed in Figures 6A, B. Consistent with the HCA results, the 42 batches of *Taxus* leaf samples divided into six categories. The samples were well separated, indicating differences in the contents of the 24 analytes in the leaves of different *Taxus* species (Figure 6A). The main components of the PCA model can predict 92.8% of the changes in the original dataset (R^2^X [cum] = 0.928), and 73.4% of the cumulative prediction rate was predicted in the model through cross-fold validation (Q^2^ [cum] = 0.734, Figure 6B). Therefore, this model can represent the information of the original data, and the obtained results are scientific and effective. In the next step, we used OPLS-DA to identify the chemical components that distinguish the six *Taxus* species.

OPLS-DA is a supervised discriminant analysis method that can be used to find variables that cause differences between samples (Li et al., 2021; Wang et al., 2022). By using PCA, we further searched for the chemical components that distinguish the different *Taxus* species. Once again, we imported the content determination results of the 24 analytes in the 42 batches of *Taxus* leaf samples into SIMCA 14.1 for OPLS-DA. The OPLS-DA scores and the variable importance predictive values (VIPs) of the 24 analytes are presented in Figures 6C, D. The established OPLS-DA model can predict 97.4% of the information in the X matrix (R^2^X [cum] = 0.974) and 87.6% of the information in the Y matrix (R^2^Y [cum] = 0.876). Cross-validation (Q^2^ [cum] = 0.854) revealed that our model is stable and has good prediction ability. The OPLS-DA score plot was similar to the PCA score plot, which divided the six *Taxus* species into six categories. This result also verified that their compositions were different (Figure 6C). Two hundred permutation tests were used for the internal validation of the model to prevent overfitting. The *R* ^2^ and *Q* ^2^ values of the six varieties were greater than 0.9 and were higher than the left (negative) of the *R* ^2^ and *Q* ^2^ values, indicating that the model and results are reliable without overfitting (Supplementary Figure S5). Therefore, VIP was used to further analyze the differential components of the six *Taxus* species (Figure 6D). The VIP indicates the contribution of each analyte to distinguishing samples, and the variables with substantial contribution to grouping and VIPs greater than 1.0 were screened as the index (Giuliani, 2017). The following analytes with VIPs greater than 1.0 can be used to distinguish the six different *Taxus* species: 10-DAB, 10-DAT, ARO, GK, BAC, GC, TAX, TAXOL, QC, EGC, 7-xyl-10-DAT, CE, C, and RT. The above analytes can be applied as potential index components to distinguish the quality of the six different *Taxus* species*.*

### 3.7 RF

**FIGURE 7:**
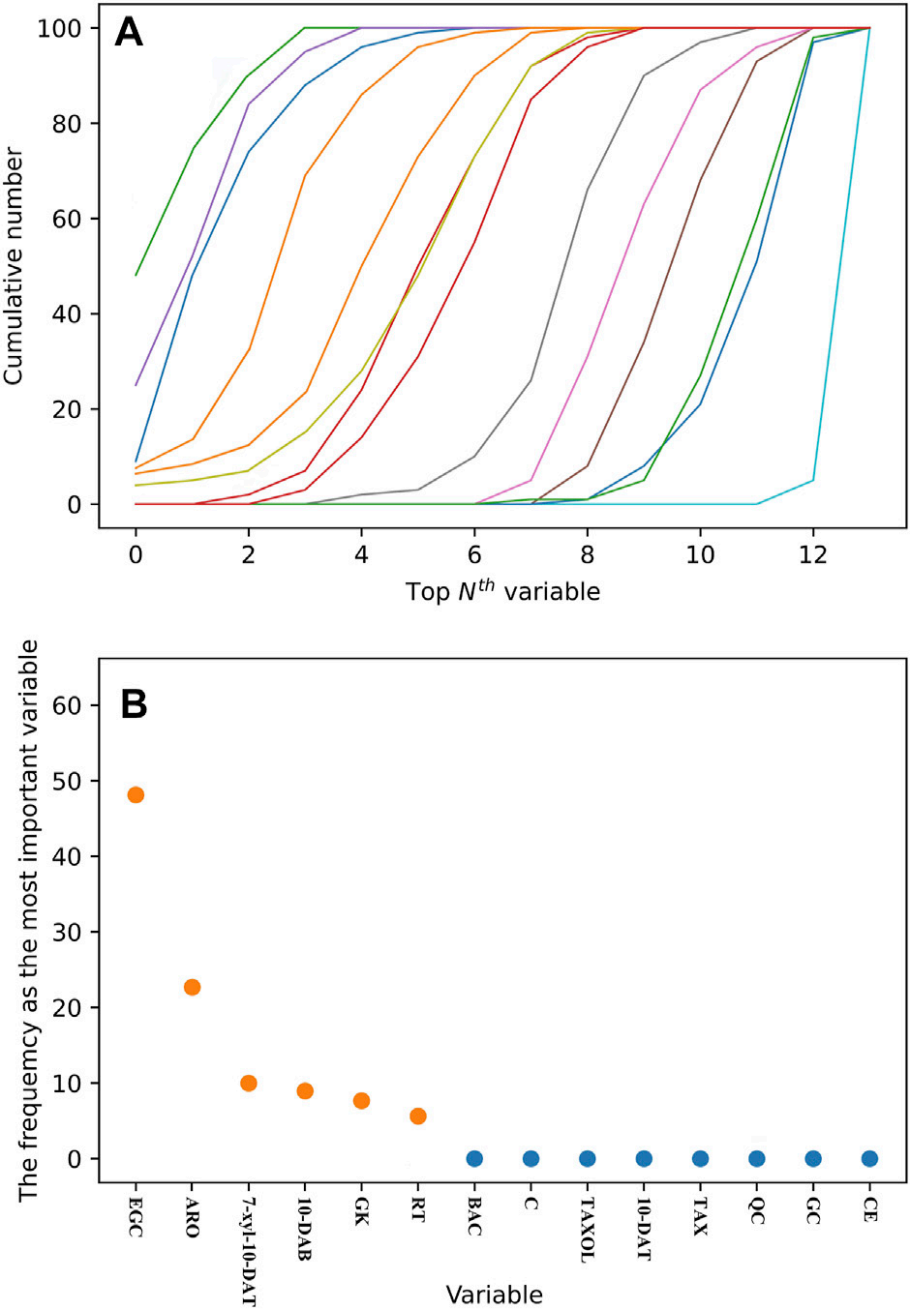
Cumulative number of each variable in the top *N*th of 100 RF iterative modeling **(A)**. Frequency of the variable as the most important parameter in 100 RF iterative modeling **(B)**.

The 14 analytes selected by OPLS-DA can be utilized as the chemical components distinguishing the six *Taxus* species. However, the detection of numerous analytes requires great cost and time. In addition, the data scaling method has a strong effect on the model. RF will not excessively scale the data under the condition of mutual comparison (Zhang et al., 2020). Additional stable components were simplified to simplify the model and distinguish the six *Taxus* species. The RF model was selected for 100 iterations, ranking the importance of the 14 analytes each time. The cumulative times of each variable in the top *N*th positions were obtained in accordance with the importance ranking of the variables in 100 RF model iterations. In Figure 7A, each line represents an analyte, and the line on the left has high characteristic importance, whereas the line on the right has the opposite characteristics. Afterward, the frequency of the variable that is the most important parameter in the 100-iteration modeling process was calculated (Figure 7B). The variable with the highest frequency was EGC, which accumulated 48 times, indicating that this analyte has a remarkable role in distinguishing the six *Taxus* species. Among other analytes, ARO accumulated 23 times, 7-xyl-10-DAT accumulated nine times, 10-DAB accumulated eight times, GK accumulated seven times, and RT accumulated five times. Therefore, we selected EGC, ARO, 7-xyl-10-DAT, 10-DAB, GK and RT to establish the model and evaluate the feasibility of simplifying the model through FDA.

### 3.8 External validation by FDA

FDA is a typical pattern discrimination method. We employed FDA to verify the feasibility of classifying six *Taxus* varieties by using the six analytes, namely, EGC, ARO, 7-xyl-10-DAT, 10-DAB, GK, and RT, selected by RF. The discriminant function was produced in accordance with the content data of the six analytes in the 42 sample batches, as follows: Y(*T. mairei*) = 0.338 7-xyl-10-DAT + 0.064 GK + 0.027 RT −0.008 ARO + 0.130 10-DAB + 0.006 EGC −68.371; Y(*T. chinensis*) = 0.317 7-xyl-10-DAT + 0.263 GK + 0.065 RT −0.072 ARO + 0.192 10-DAB + 0.007 EGC −389.484; Y(*T. yunnanensis*) = 1.065 7-xyl-10-DAT + 0.003 GK + 0.044 RT −0.080 ARO + 0.790 10-DAB −0.016 EGC −512.021; Y(*T. wallichiana*) = 0.005 7-xyl-10-DAT + 0.002 GK + 0.001 RT + 0.000 ARO + 0.002 10-DAB + 0.001 EGC −1.885; Y(*T. cuspidata*) = 0.010 7-xyl-10-DAT + 0.043 GK − 0.002 RT + 0.683 ARO + 0.005 10-DAB + 0.024 EGC −187.611; Y(*T. media*) = 0.042 7-xyl-10-DAT + 0.057 GK + 0.015 RT + 0.480 ARO + 0.044 10-DAB + 0.065 EGC −172.673. Additionally, we selected samples S43–S51 outside the model and determined only the content of the six analytes (the content data results are shown in Table 8) for external validation. The samples of the established model and those outside the model based on the discriminant function of the linear combination of predictive variables were correctly discriminated and classified (Table 9), thus verifying the accuracy and practicality of the simplified model. We compared the differences in the chemical structures of the six analytes from *Taxus* species. 7-Xyl-10-DAT and 10-DAB were taxoids, and their distinguishing radical pharmacophores were hydroxyl and xylose groups; EGC was a flavanol, and its distinguishing radical pharmacophore was hydroxyl group; ARO was a dihydroflavone, and its distinguishing radical pharmacophore was C-ring α-hydroxyl and B-ring hydroxyl groups; GK was a biflavone, and its distinguishing radical pharmacophores were methoxy and hydroxyl groups; RT was a flavonol, and its distinguishing radical pharmacophores was rutinoside group. In Figure 1, circles mark the distinguishing radical pharmacophores of the compounds.

**TABLE 8:** Externally validated assay data for the FDA model.

NO.	Content of analytes (μg/g)					
	7-xyl-10-DAT	GK	RT	ARO	10-DAB	EGC
S43	139.83	478.94	1776.45	49.35	18.81	593.31
S44	194.27	399.72	1,392.32	36.53	15.74	414.69
S45	64.27	1,603.37	4,519.25	78.05	110.93	658.84
S46	348.89	150.16	904.26	16.19	808.69	331.27
S47	340.15	162.83	923.08	14.66	899.13	274.94
S48	3.36	9.07	113.73	3.45	3.08	30.15
S49	3.61	472.25	707.18	499.36	55.81	115.91
S50	3.74	445.12	738.41	513.78	43.83	136.73
S51	38.82	482.04	2034.36	340.59	36.94	1801.36

**TABLE 9:** FDA model classification results.

		Predicted group membership						Total	Accuracy (%)
		*T. mairei*	*T. chinensis*	*T. yunnanensis*	*T. wallichiana*	*T. cuspidata*	*T. media*		
Geographical origin	*T. mairei*	15	0	0	0	0	0	15	100
	*T. chinensis*	0	5	0	0	0	0	5	
	*T. yunnanensis*	0	0	5	0	0	0	5	
	*T. wallichiana*	0	0	0	5	0	0	5	
	*T. cuspidata*	0	0	0	0	6	0	6	
	*T. media*	0	0	0	0	0	6	6	
Cross-validated	*T. mairei*	15	0	0	0	0	0	15	100
	*T. chinensis*	0	5	0	0	0	0	5	
	*T. yunnanensis*	0	0	5	0	0	0	5	
	*T. wallichiana*	0	0	0	5	0	0	5	
	*T. cuspidata*	0	0	0	0	6	0	6	
	*T. media*	0	0	0	0	0	6	6	
External-validated	*T. mairei*	2	0	0	0	0	0	2	100
	*T. chinensis*	0	1	0	0	0	0	1	
	*T. yunnanensis*	0	0	2	0	0	0	2	
	*T. wallichiana*	0	0	0	1	0	0	1	
	*T. cuspidata*	0	0	0	0	2	0	2	
	*T. media*	0	0	0	0	0	1	1	

## 4 Conclusion

We established a UPLC–MS/MS method for the simultaneous determination of 24 analytes in the leaves of six *Taxus* species. Methodological verification demonstrated that our method can be used for rapid and accurate analysis. The results of UPLC–MS/MS combined with chemometric analysis revealed that six analytes, namely, EGC, ARO, 7-xyl-10-DAT, 10-DAB, GK, and RT, could be used to distinguish the six *Taxus* species for quality control. This chemical model can be used as a simple way to distinguish six different *Taxus* species. The effect of the varieties of these species on the effectiveness of use should be considered, and additional work needs to be done to evaluate and verify the analysis results of our study.

## Data Availability

The original contributions presented in the study are included in the article/Supplementary Material, further inquiries can be directed to the corresponding authors.
