# Supplementary material for: Quality evaluation of compounds in leaves of six Taxus species based on UPLC-MS/MS and chemometrics
Source: Front Chem. 2023 May 31;11:1193188. doi: 10.3389/fchem.2023.1193188 (PMC10264637; doi:10.3389/fchem.2023.1193188)
Supplement: Supplementary file 1 [file DataSheet1.pdf]

Supplementary Material

Quality evaluation of compounds in leaves of six Taxus species based on UPLC-MS/MS and chemometrics

Qingzhu Cai[1†], Qiang Song[2†], Kunxia Jiang[1], Yao Lin[1], Ying Zhang[1], Jirong Zhang[1], Shuqing Lin[1], Lina Huang[1], Qihuang Xue[3], Zehao Huang[1,*], Wen Xu[1,*], Wei Xu[1], Mun Fei Yam[1,4*]

[1] College of Pharmacy, Fujian University of Traditional Chinese Medicine, Fuzhou, China

[2] Department of Pharmacy, Fujian Provincial Hospital, Shengli Clinical Medical College of Fujian Medical University, Fuzhou 350001, P. R. China

[3] Fujian South Pharmaceutical Co.,Ltd, Sanming, China

[4] Department of Pharmacology, School of Pharmaceutical Sciences, Universiti Sains Malaysia, Penang, Malaysiak

*** Correspondence:**

Zehao Huang huangzehao@fudan.edu.cn;Wen Xu 2012029@fjtcm.edu.cn; Mun Fei Yam yammunfei@yahoo.my

†These authors have contributed equally to this work

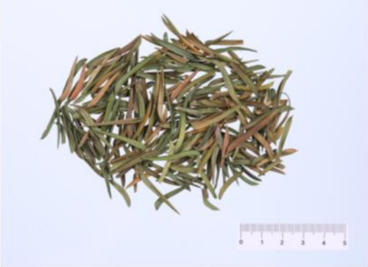

S1 Mingxi, Fujian

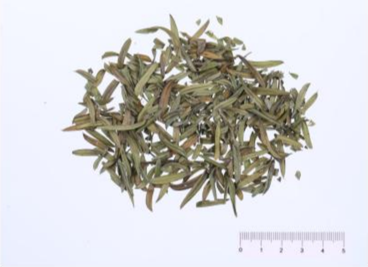

S2 Anxi, Fujian

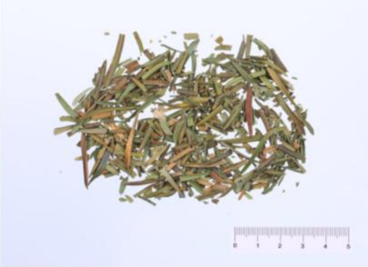

S3 Mingxi, Fujian

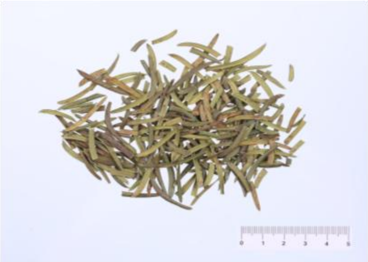

S4 Mingxi, Fujian

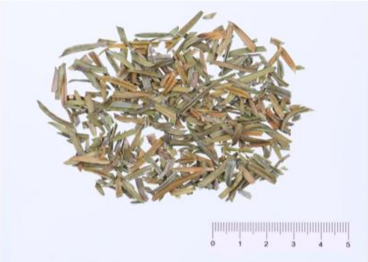

S5 Huzhou, Zhejiang

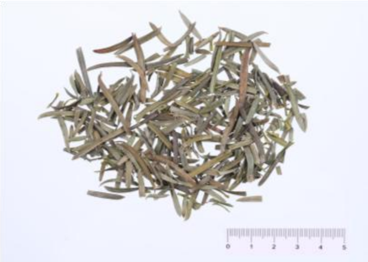

S6 Yichun, Jiangxi

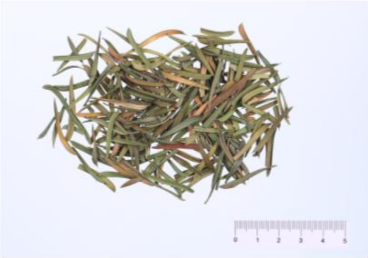

S7 Minhou, Fujian

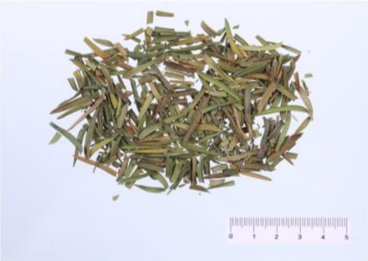

S8 Minhou, Fujian

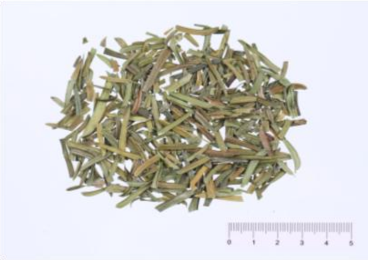

S9 Mingxi, Fujian

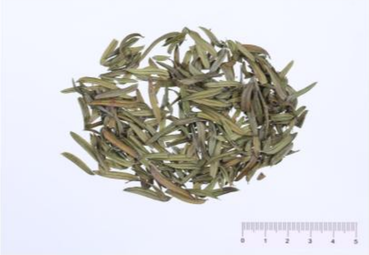

S10 Minqing, Fujian

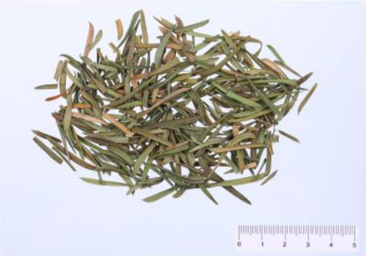

S11 Mingxi, Fujian

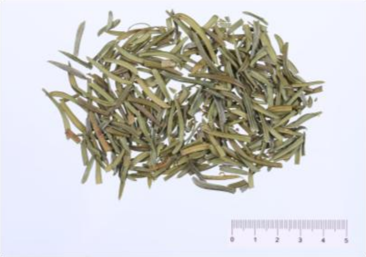

S12 Ganzhou, Jiangxi

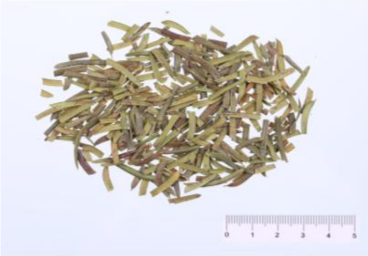

S13 Yongzhou, Hunan

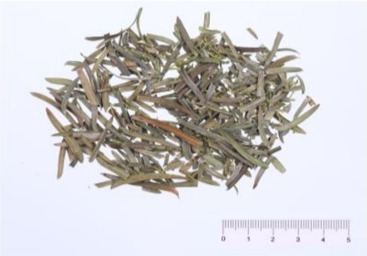

S14 Zhangzhou, Fujain

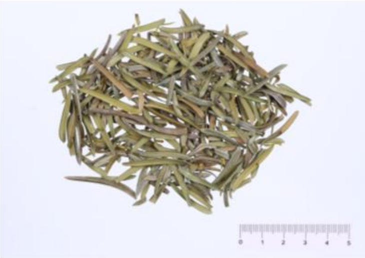

S15 Qiandongnan, Guizhou

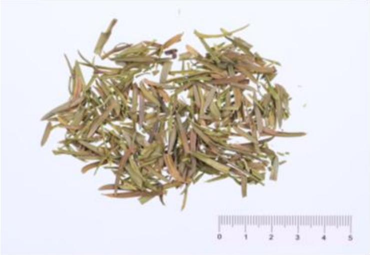

S16 Tianshui, Gansu

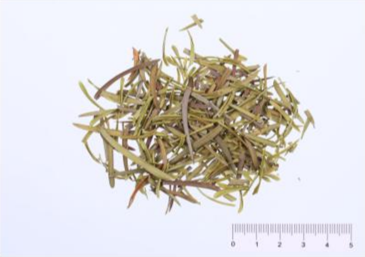

S17 Weinan, Shanxi

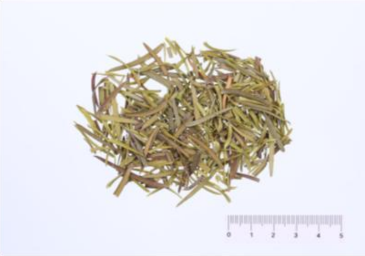

S18 Weinan, Shanxi

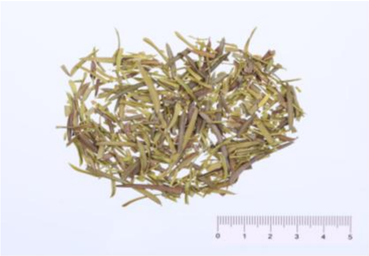

S19 Weinan, Shanxi

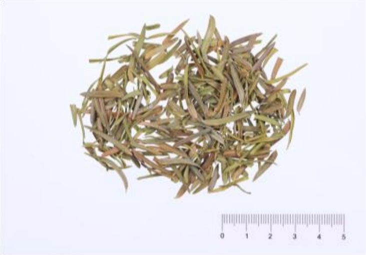

S20 Tianshui, Gansu

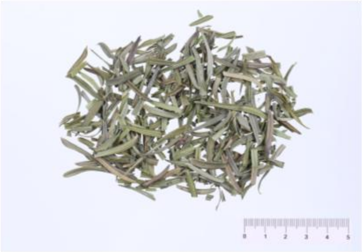

S21 Kunming, Yunnan

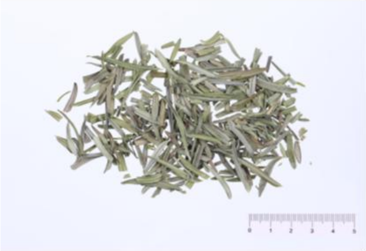

S22 Kunming, Yunnan

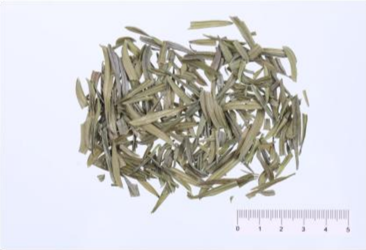

S23 Yongtai, Fujian

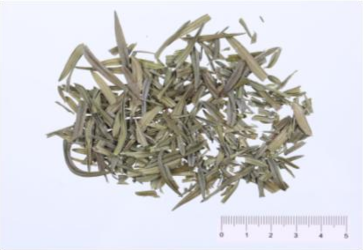

S24 Yongtai, Fujian

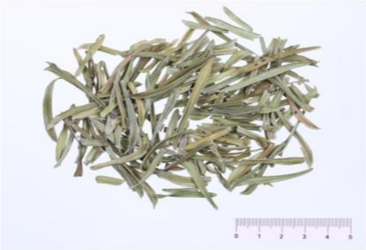

S25 Dali, Yunnan

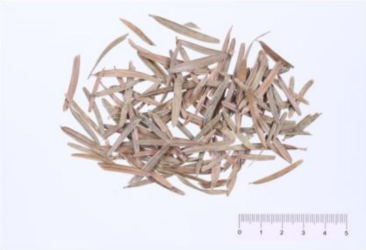

S26 Hami, Xinjiang

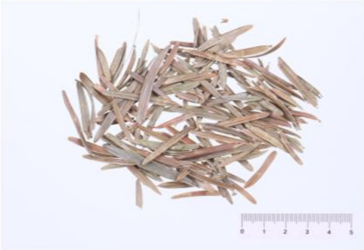

S27 Hami, Xinjiang

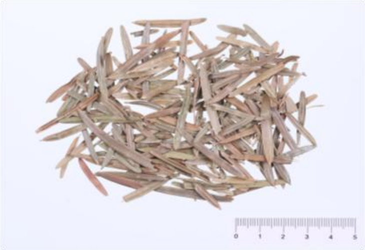

S28 Hami, Xinjiang

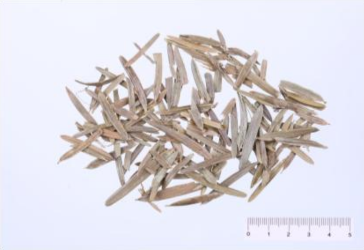

S29 Daqing, Heilongjiang

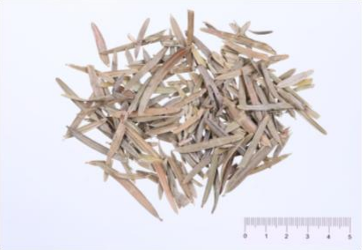

S30 Daqing, Heilongjiang

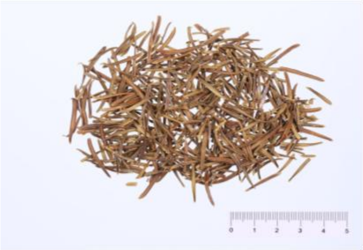

S31 Mianyang, Sichuan

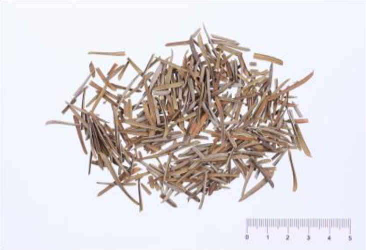

S32 Changchun, Jilin

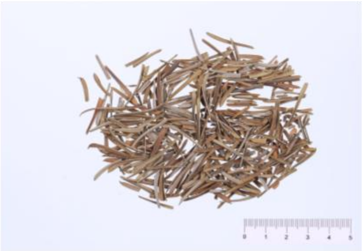

S33 Tonghua, Jilin

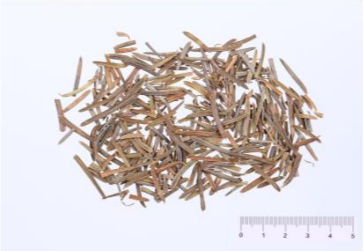

S34 Anshan, Liaoning

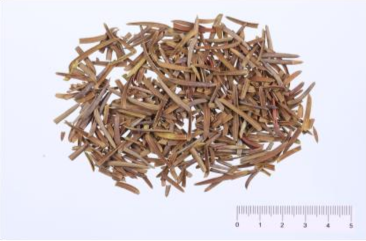

S35 Mudanjiang, Heilongjiang

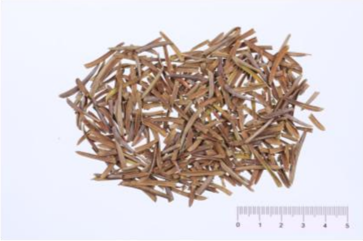

S36 Mudanjiang, Heilongjiang

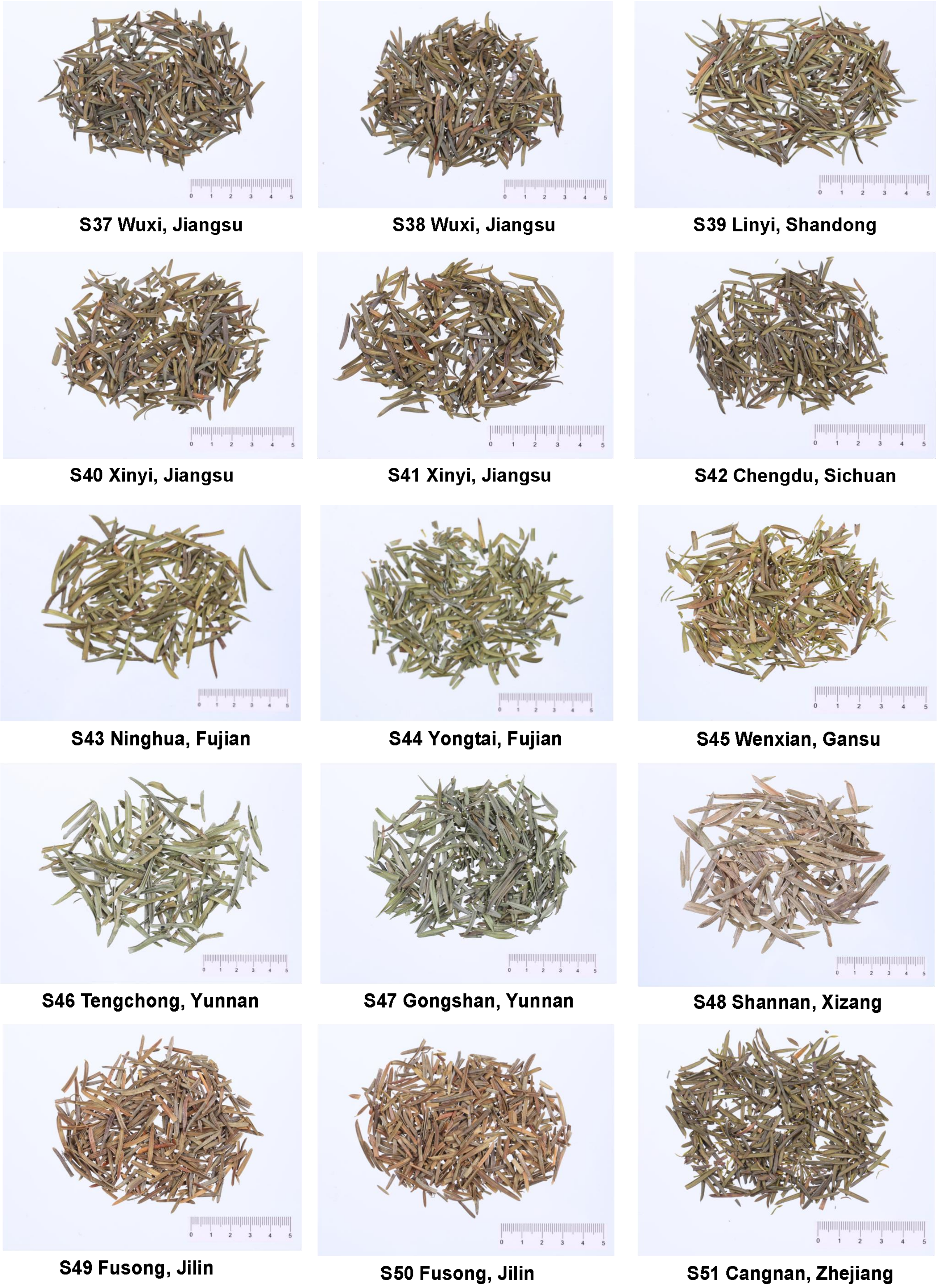

Figure S1. Fifty-one leaf samples from the six *Taxus* species.

(S43 to S51 are used for external validation of the model.)

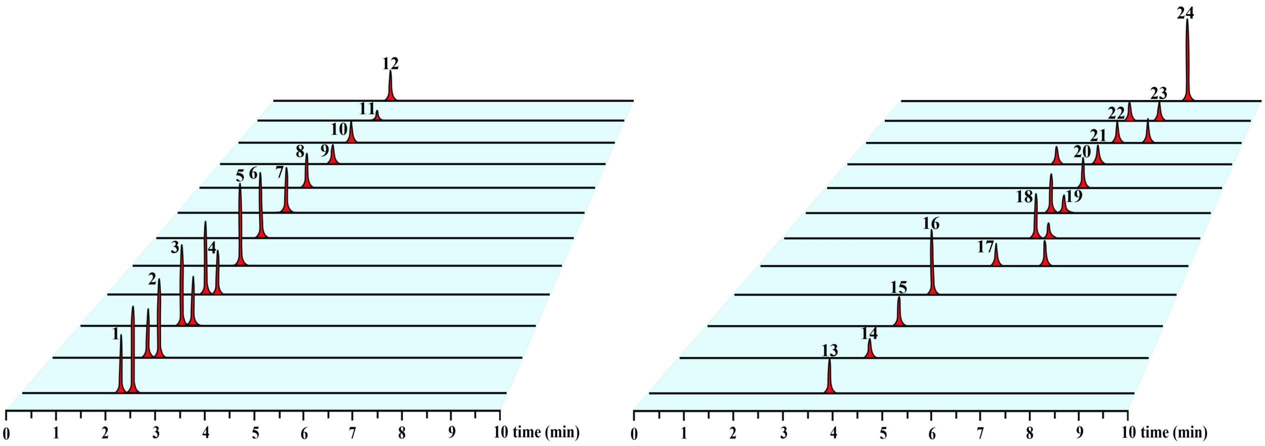

Figure S2. UPLC-MS/MS chromatograms of 24 analytes in a representative sample of *T. mairei* (S3).

1.GC, 2.EGC, 3.C, 4.EC, 5,RT, 6.IQC, 7.NFR, 8.QC, 9.TAX, 10.ARO, 11.QR, 12.10-DAB, 13.AF, 14.BAC, 15.DGK, 16.7-xyl-10-DAT, 17.10-DAT, 18.GK, 19.IGG, 20.CE, 21.7-epi-10-DAT, 22.TAXOL, 23.7-epi-TAXOL, 24.SDN.

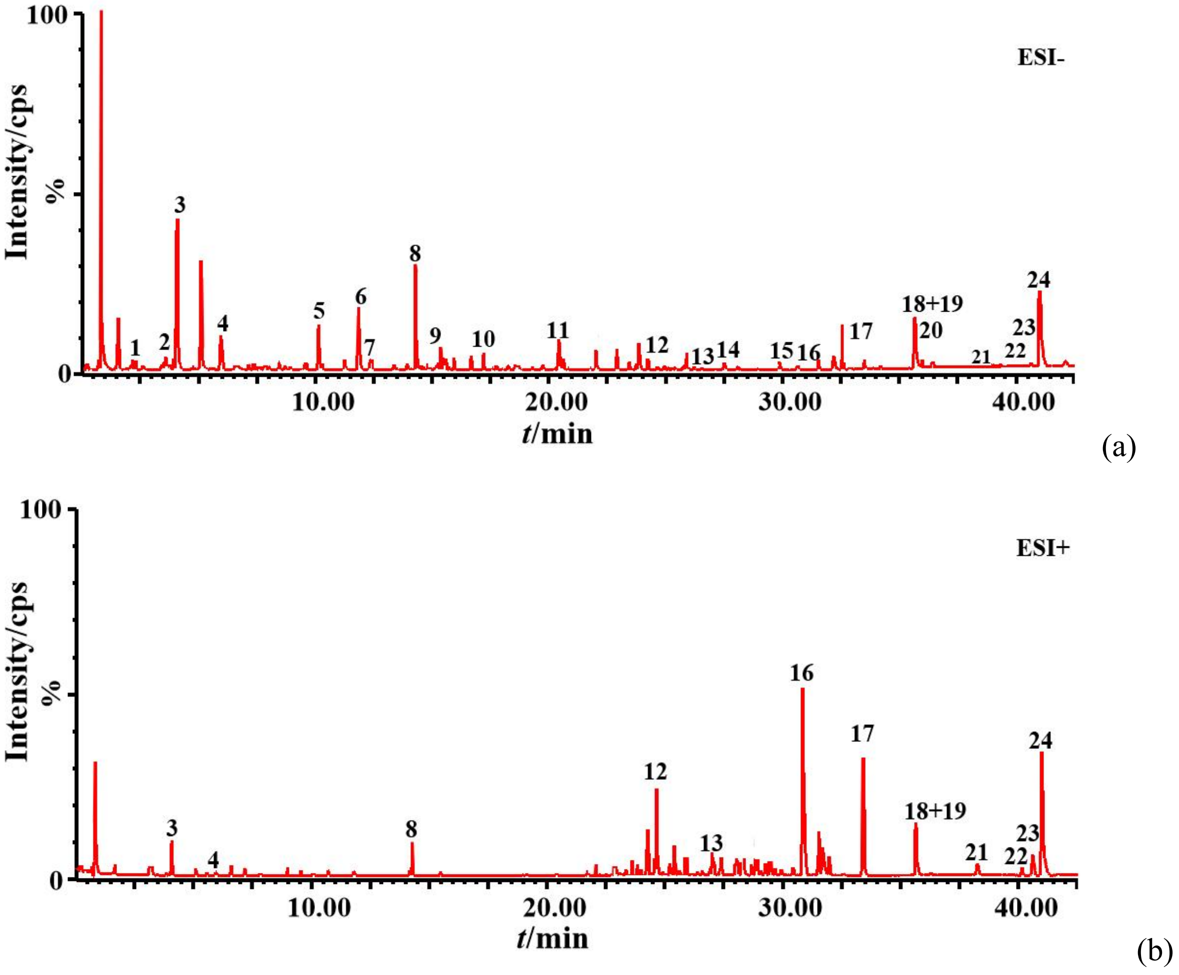

Figure S3. The total ion chromatogram of *T. mairei* obtained in the negative (a) and positive (b)

modes by HPLC-Q-TOF-MS.

Note: An ACQUITY HPLC system coupled with a Xevo XS quadrupole time of flight mass spectrometer (Waters, Milford, MA, USA) was used for characterization of chemical components in *T. mairei*. Chromatographic separation was carried out at 40 °C on a Waters CORTECS C18 column (4.6 mm * 150 mm; 2.7 μm), with 0.1% of formic acid as mobile phase A and acetonitrile as mobile phase B. Gradient elution was performed as follows: 5%-5% B for 0-0.5 min, 5%-9% B for 0.5-3 min, 9%-11% B for 3-7 min, 11%-12.5% B for 7-12 min, 12.5%-20% B for 12-20 min, 20%-36% B for 20-30 min, 36%-70% B for 30-42.5 min. The flow rate was set at 0.5 mL/min. The mass-spectrometry conditions were optimized as follows: ESI negative and positive mode, desolvent gas temperature, 500 °C; capillary voltage, -2.5 kV and +3.0 kV; source temperature, 150 °C; desolvent gas flow (N_2), 800 L/h; and cone gas flow (N_2), 50 L/h. The MS scan range was *m/z* 50-1800, and the collision energy was set at 10-55 eV.

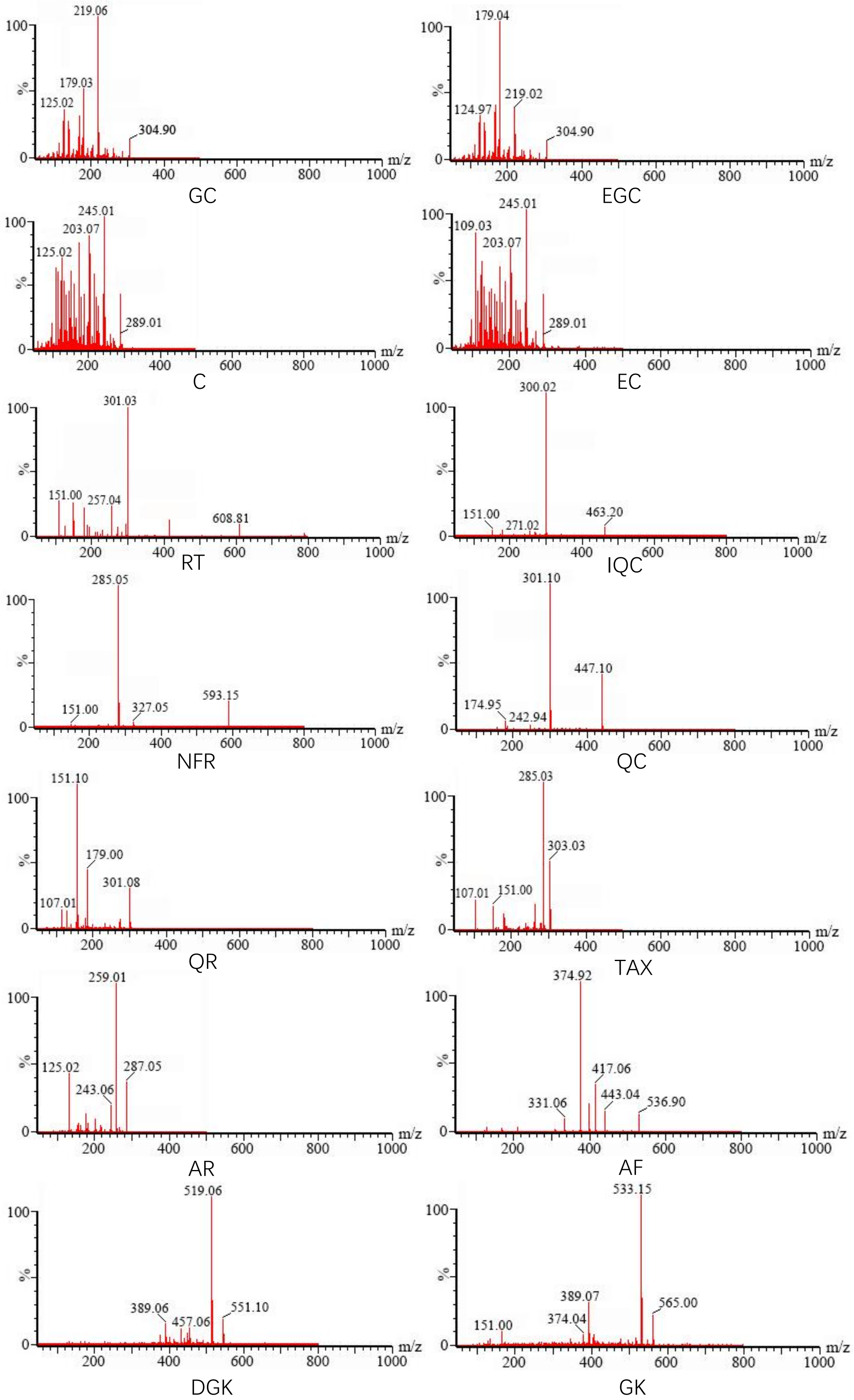
GC
219.06
179.03
125.02
304.90
EGC
179.04
124.97
219.02
304.90
C
245.01
203.07
125.02
289.01
EC
245.01
109.03
203.07
289.01
RT
301.03
151.00
257.04
608.81
IQC
300.02
151.00
271.02
463.20
NFR
285.05
151.00
327.05
593.15
QC
301.10
447.10
174.95
242.94
QR
151.10
179.00
301.08
107.01
TAX
285.03
303.03
107.01
151.00
AR
259.01
125.02
287.05
243.06
AF
374.92
417.06
443.04
331.06
536.90
DGK
519.06
389.06
457.06
551.10
GK
533.15
389.07
565.00
374.04
151.00
%
m/z

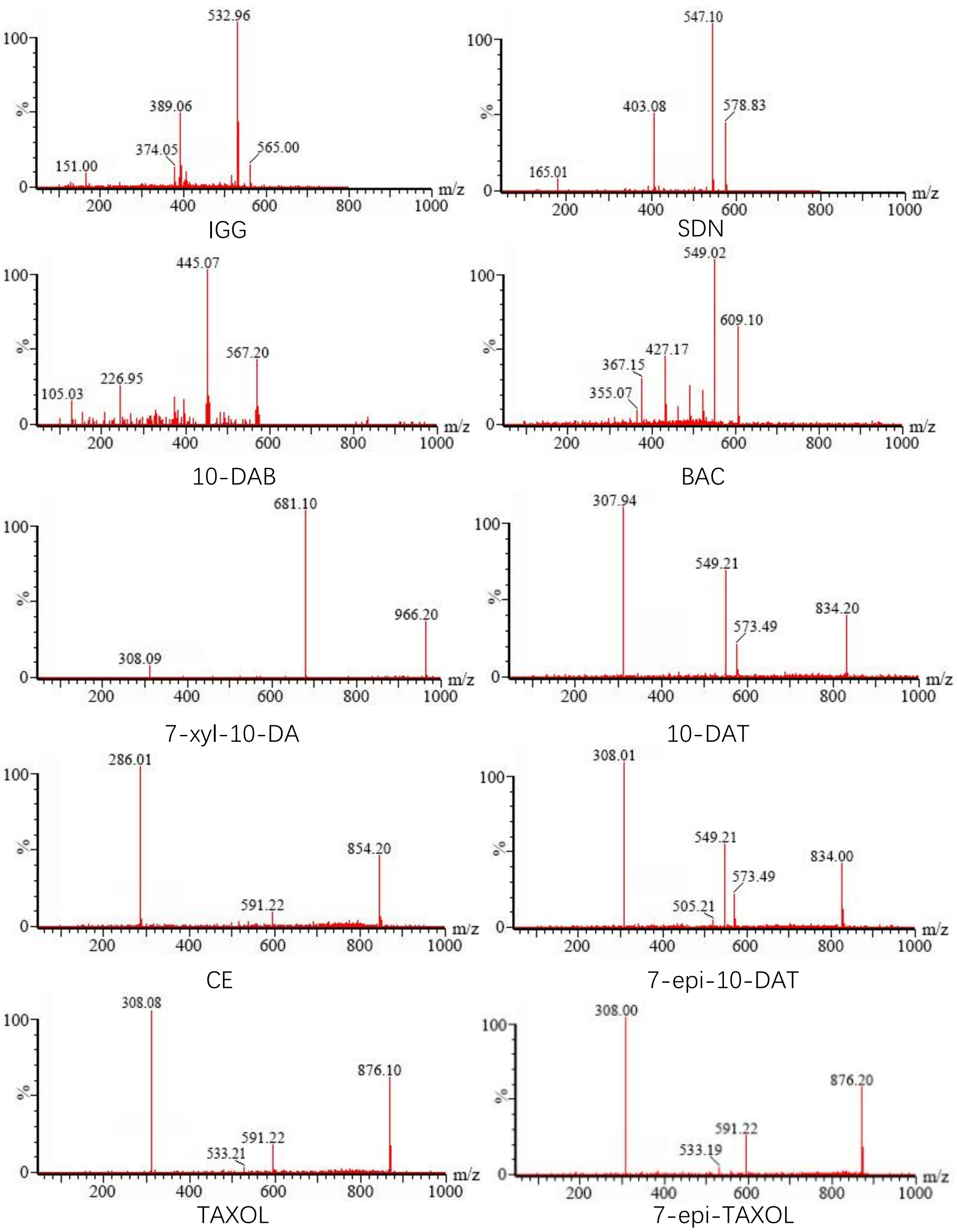

Figure S4. Mass spectrum information of 24 analyses.

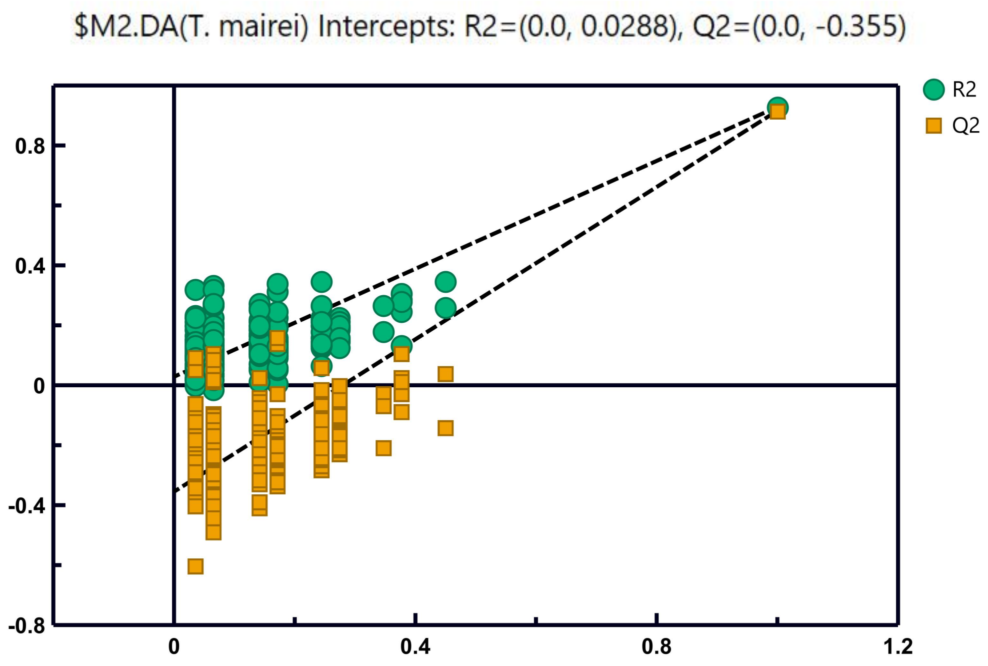
$M2.DA(T. mairei) Intercepts: R2=(0.0, 0.0288), Q2=(0.0, -0.355)
R2
Q2
0.8
0.4
0
-0.4
-0.8
0
0.4
0.8
1.2

(a)

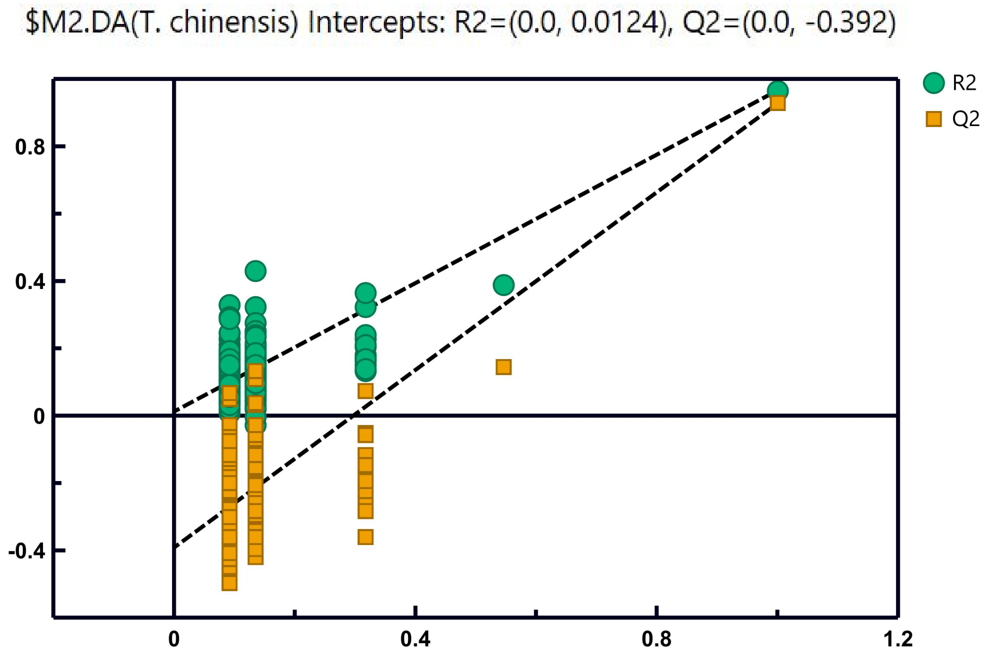
$M2.DA(T. chinensis) Intercepts: R2=(0.0, 0.0124), Q2=(0.0, -0.392)
R2
Q2
0.8
0.4
0
-0.4
0
0.4
0.8
1.2

(b)

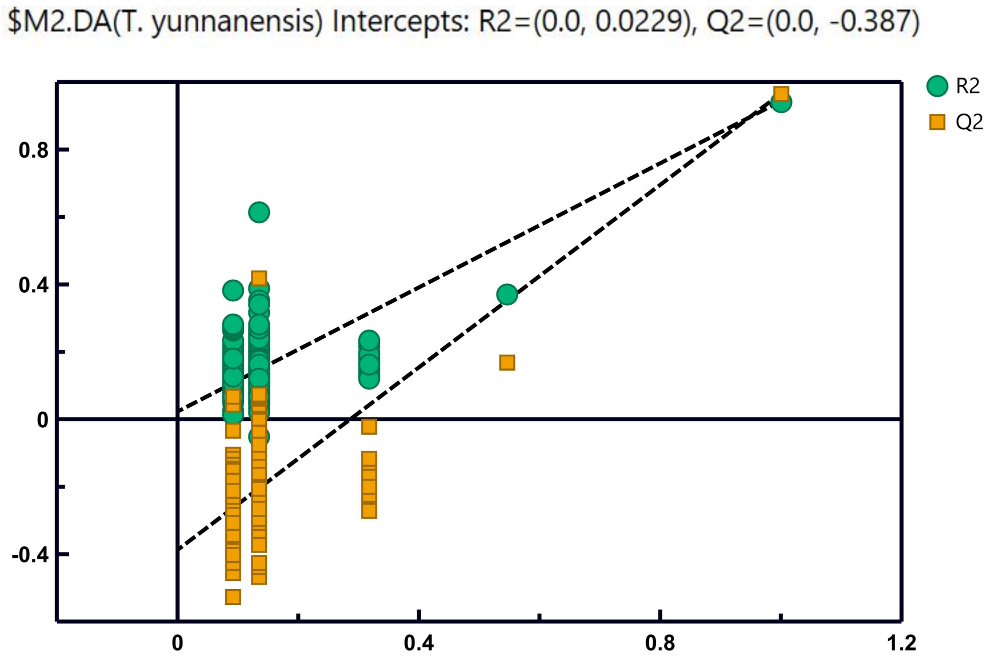
$M2.DA(T. yunnanensis) Intercepts: R2=(0.0, 0.0229), Q2=(0.0, -0.387)
R2
Q2
0.8
0.4
0
-0.4
0
0.4
0.8
1.2

(c)

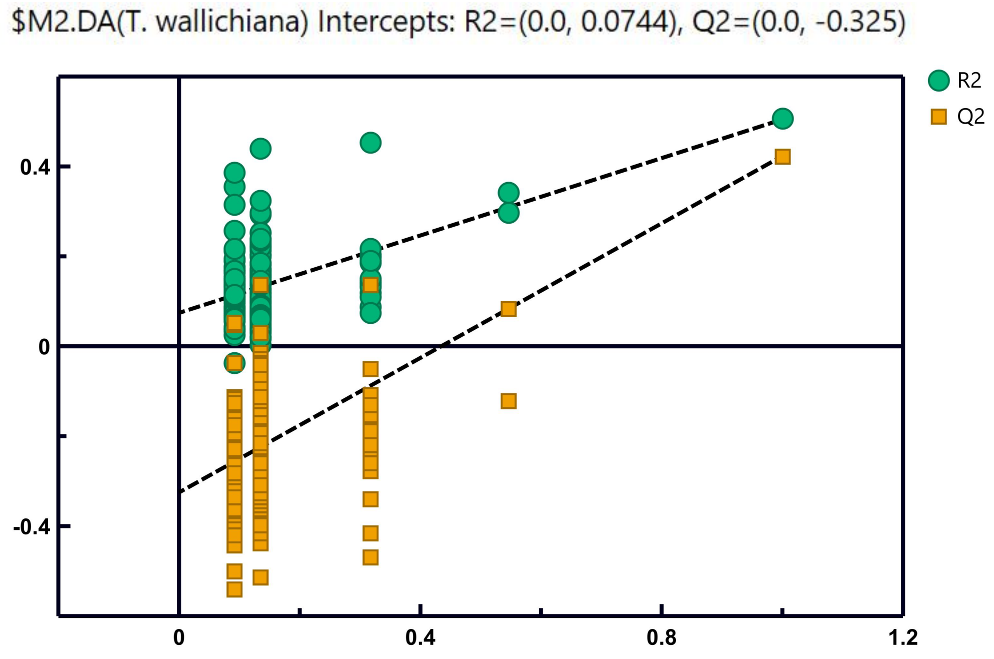
$M2.DA(T. wallichiana) Intercepts: R2=(0.0, 0.0744), Q2=(0.0, -0.325)
R2
Q2
0.4
0
-0.4
0
0.4
0.8
1.2

(d)

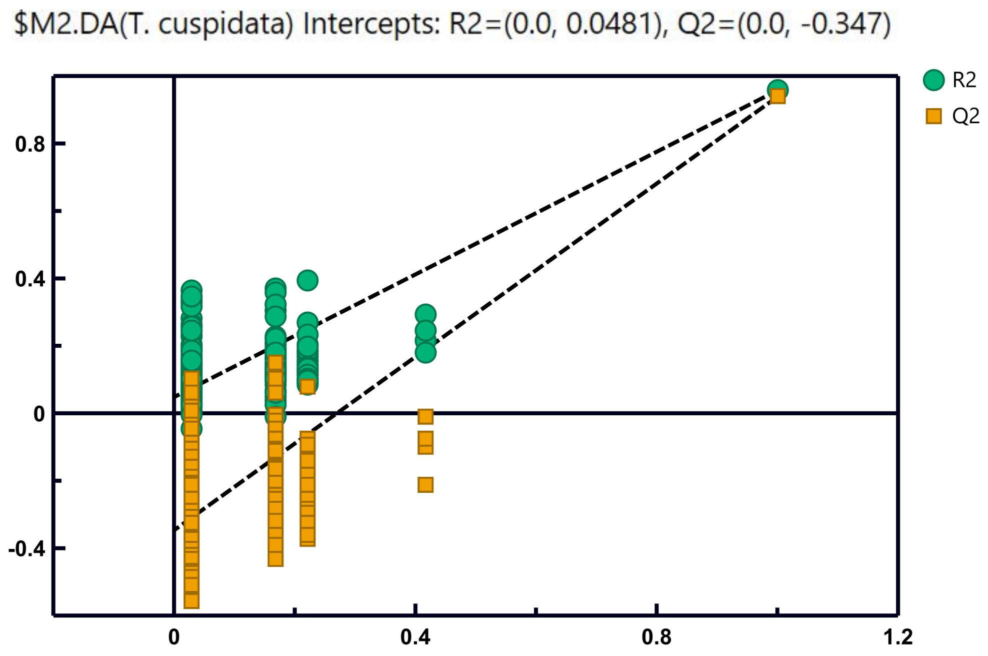

(e)

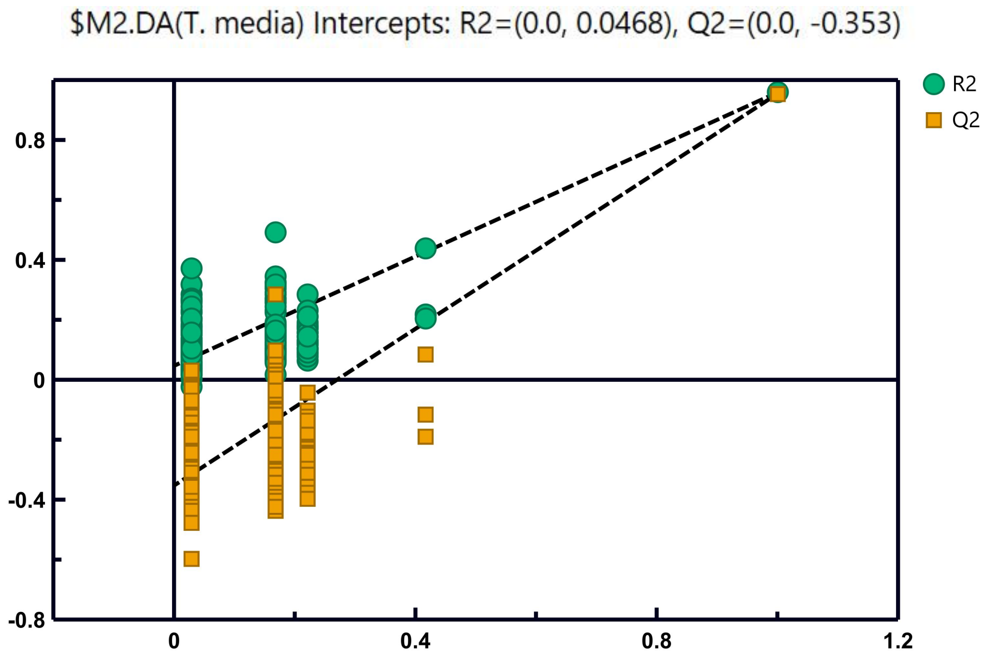

(f)

Figure S5. Two hundred permutation tests of 6 varieties.

a: *T. mairei*, b: *T. chinensis*, c: *T. yunnanensis*, d: *T. wallichiana*, e: *T. cuspidata*, f: *T. media.*

Table S1. Compares the results of the present study with those of other scholars.

Literature	Sample types	Quantitative analytes	Method time	Consumption of mobile phase solvent	LOD		LOQ	
					Compounds	(ng/mL)	Compounds	(ng/mL)
**This article**	Six *Taxus* species (*T. mairei*, *T. chinensis*, *T. yunnanensis*, *T. wallichiana*, *T. cuspidata*, *T. media.*)	eight taxoids (10-DAB, BAC, 7-xyl-10-DAT, 10-DAT, CE, 7-epi-10-DAT, TAXOL, 7-epi-TAXOL);<br>four flavanols (GC, EGC, C, EC);<br>five flavonols (RT, IQC, NFR, QC, QR);<br>two dihydroflavones (TAX, ARO);<br>five biflavones (AF, DGK, GK, IGG, SDN).	10.5 minutes	2.625 mL	GC	1.28	GC	2.56
					EGC	1.53	EGC	3.05
					C	1.23	C	2.45
					EC	0.48	EC	0.96
					RT	0.53	RT	1.06
					IQC	1.23	IQC	2.46
					NFR	1.23	NFR	2.46
					QC	1.36	QC	2.73
					TAX	0.58	TAX	1.16
					ARO	0.49	ARO	0.98
					QR	0.52	QR	1.05
					10-DAB	0.52	10-DAB	1.05
					AF	0.47	AF	0.94
					BAC	0.56	BAC	1.12
					DGK	0.50	DGK	1.01
					7-xyl-10-DAT	0.55	7-xyl-10-DAT	1.11
					10-DAT	0.59	10-DAT	1.17
					GK	1.32	GK	2.64
					IGG	0.97	IGG	1.94
					CE	0.57	CE	1.14
					7-epi-10-DAT	0.49	7-epi-10-DAT	0.98
					TAXOL	1.00	TAXOL	1.99
					7-epi-TAXOL	0.59	7-epi-TAXOL	1.19

					SDN	0.53	SDN	1.05
Analysis of the annual variation of five flavonoids contents in *Taxus chinensis* var. *mairei* in Hubei Zhongxiang	One *Taxus* specie (*T. mairei*)	Five flavonoids (RT, QR, AF, IGG, SDN)	40 minutes	40 mL	/		/	
Determination and analysis of seven Taxanes in different *Taxus* species by HPLC method.	Three *Taxus* species (*T. chinensis*, *T. cuspidata*, *T. media.*)	Seven taxoids (10-DAB, BAC, 7-xyl-10-DAT, 10-DAT, CE, 7-epi-10-DAT, TAXOL)	40 minutes	40 mL	Compounds	(μg/mL)	Compounds	(μg/mL)
					10-DAB	0.10	10-DAB	0.30
					BAC	0.09	BAC	0.27
					7-xyl-10-DAT	0.08	7-xyl-10-DAT	0.25
					10-DAT	0.07	10-DAT	0.21
					CE	1.10	CE	3.30
					7-epi-10-DAT	0.09	7-epi-10-DAT	0.28
					TAXOL	1.00	TAXOL	3.00
Determination of paclitaxel and other six taxoids in *Taxus* species by high-performance liquid chromatography-tandem mass spectrometry	Three *Taxus* species (*T. mairei*, *T. cuspidata*, *T. media.*)	Seven taxoids (10-DAB, BAC, 7-xyl-10-DAT, 10-DAT, CE, 7-epi-10-DAT, TAXOL)	45 minutes	45 mL	Compounds	(ng/mL)	Compounds	(ng/mL)
					10-DAB	10	10-DAB	32
					BAC	4	BAC	14
					7-xyl-10-DAT	8	7-xyl-10-DAT	26
					10-DAT	4	10-DAT	14
					CE	6	CE	20

					TAXOL	10	TAXOL	32
					7-epi-TAXOL	8	7-epi-TAXOL	26
Simultaneous determination of taxoids and flavonoids in twigs and leaves of three *Taxus* species by UHPLC-MS/MS	Three *Taxus* species (*T. mairei*, *T. cuspidata*, *T. media.*)	Seven taxoids （10-DAB, BAC, 7-xyl-10-DAT, 10-DAT, CE, TAXOL, 7-epi-TAXOL）and Seven flavonoids (IQC, QC, QR, DGK, GK, IGG, SDN)	5 minutes	2 mL	/		Compounds<br>10-DAB<br>BAC<br>7-xyl-10-DAT<br>10-DAT<br>CE<br>TAXOL<br>7-epi-TAXOL<br>IQC<br>QC,<br>QR<br>DGK<br>GK<br>IGG<br>SDN	(ng/mL)<br>0.17<br>0.09<br>0.16<br>0.05<br>0.16<br>0.03<br>0.20<br>0.49<br>0.13<br>1.66<br>0.12<br>0.01<br>0.03<br>0.08

***Table S2**. Qualitative analysis of chemical constituents in T. mairei.*

NO.	t_R *(min)*	*Detected* m/z	*Expected* m/z	*Error* （*ppm*）	*Fragment Ions Collected in Positive Mode*	*Molecular Formula*	*Identity*	*Classify*
1	*2.19*	*305.0663*	*305.0661*	*0.7*	*261.0760 [M-H-CO_2]$^-$, 219.0651 [M-H-CO_2-C_2H_2O]$^-$ 179.0347 [M-H-$C_6H_6O_2$]$^-$ 125.0230 [M-H-*	$C_{15}H_{14}O_7$	*GC*	*Flavanols*
2	*3.62*	*305.0663*	*305.0661*	*0.7*	*$C_9H_8O_3$]$^-$ 261.0758 [M-H-CO_2]$^-$, 219.0647 [M-H-CO_2-C_2H_2O]$^-$ 179.0342 [M-H-$C_6H_6O_2$]$^-$ 125.0232 [M-H-*	$C_{15}H_{14}O_7$	*EGC*	*Flavanols*
3	*4.10*	*289.0701*	*289.0712*	*-3.8*	*$C_9H_8O_3$]$^-$ 245.0798 [M-H-CO_2]$^-$, 203.0661 [M-H-CO_2-C_2H_2O]$^-$, 151.0374 [M-H-$C_7H_6O_3$]$^-$, 125.0231 [M-H-*	$C_{15}H_{14}O_6$	*C*	*Flavanols*
4	*5.96*	*289.0701*	*289.0712*	*-3.8*	*$C_9H_8O_3$]$^-$ 245.0799 [M-H-CO_2]$^-$, 203.0664 [M-H-CO_2-C_2H_2O]$^-$, 151.0376 [M-H-$C_7H_6O_3$]$^-$, 125.0233 [M-H-*	$C_{15}H_{14}O_6$	*EC*	*Flavanols*
5	*10.15*	*609.1429*	*609.1456*	*-4.4*	*$C_9H_8O_3$]$^-$ 301.0347 [M-H-Glc-Rha]$^-$, 257.0452 [M-H-Glc-Rha-CO_2]$^-$, 151.0035 [M-H-*	$C_{27}H_{30}O_{16}$	*RT*	*Flavonols*
6	*11.86*	*463.0864*	*463.0877*	*-2.8*	*$C_{20}H_{26}O_{12}$]$^-$ 300.0320 [M-H-Glc]$^-$, 178.9964 [M-H-$C_{13}H_{16}O_7$]$^-$, 151.0028 [M-H-*	$C_{21}H_{20}O_{12}$	*IQC*	*Flavonols*
7	*12.38*	*593.1509*	*593.1506*	*0.5*	*$C_{14}H_{16}O_8$]$^-$ 285.0400 [M-H-Glc-Rha]$^-$, 151.0024 [M-H-Glc-Rha-*	$C_{27}H_{30}O_{15}$	*NFR*	*Flavonols*
8	*14.29*	*447.0906*	*447.0927*	*-4.7*	*$C_8H_6O_2$]$^-$ 301.0323 [M-H-Rha]$^-$, 179.0608 [M-H-$C_{13}H_{16}O_6$]$^-$, 151.0043 [M-H-$C_{14}H_{16}O_7$]$^-$*	$C_{21}H_{20}O_{11}$	*QC*	*Flavonols*

9	15.36	303.0510	303.0505	1.6	285.0392 $[M-H-H_2O]^-$, 151.0036 $[M-H-C_8H_8O_3]^-$, 107.0138 $[M-H-C_8H_8O_3-CO_2]^-$	$C_{15}H_{12}O_7$	TAX	Dihydroflavones
10	17.25	287.0551	287.0556	-1.7	259.0596 $[M-H-CO]^-$, 243.0683 $[M-H-CO_2]^-$, 201.0554 $[M-H-CO_2-C_2H_2O]^-$	$C_{15}H_{12}O_6$	ARO	Dihydroflavones
11	20.45	301.0346	301.0348	-0.7	273.0362 $[M-H-CO]^-$, 178.9969 $[M-H-C_6H_2O_3]^-$, 151.0015 $[M-H-C_8H_6O_3]^-$, 107.0128 $[M-H-C_8H_6O_3-CO_2]^-$	$C_{15}H_{10}O_7$	QR	Flavonols
12	24.67	567.2201	567.2206	-0.9	531.6315 $[M+Na-2H_2O]^+$, 445.0602 $[M+Na-2H_2O-CH_3COOH]^+$	$C_{29}H_{36}O_{10}$	10-DAB	Taxoids
13	27.02	537.0795	537.0787	-5.0	443.0389 $[M-H-C_5H_2O_2]^-$, 417.0619 $[M-H-C_7H_4O_2]^-$, 399.0490 $[M-H-C_7H_4O_2-H_2O]^-$, 375.0494 $[M-H-C_9H_6O_3]^-$	$C_{30}H_{18}O_{10}$	AF	Biflavones
14	27.51	609.2316	609.2312	0.7	549.2018 $[M+Na-CH_3COOH]^+$, 427.1650 $[M+Na-CH_3COOH-PhCOOH]^+$, 367.1466 $[M+Na-2CH_3COOH-PhCOOH]^+$,	$C_{31}H_{38}O_{11}$	BAC	Taxoids
15	29.89	551.0994	551.0978	2.9	519.0698 $[M-H-CH_3OH]^-$, 475.0842 $[M-H-CH_3OH-CO_2]^-$, 457.0732 $[M-H-CH_3OH-CO_2-H_2O]^-$, 389.0673 $[M-H-C_9H_6O_3]^-$	$C_{31}H_{20}O_{10}$	DGK	Biflavones
16	30.88	966.3520	966.3524	-0.4	681.2596 $[M+Na-C_{16}H_{15}NO_4]^+$, 308.0893$[M+Na-C_{34}H_{42}O_{13}]^+$	$C_{50}H_{57}NO_{17}$	7-xyl-10-DAT	Taxoids
17	33.46	834.3089	834.3102	-1.6	549.2124$[M+Na-C_{16}H_{16}NO_4]^+$, 308.0833 $[M+Na-C_{29}H_{34}O_9]^+$	$C_{45}H_{49}NO_{13}$	10-DAT	Taxoids
18	35.58	565.1160	565.1135	4.4	533.0857 $[M-H-CH_3OH]^-$, 389.0624 $[M-H-CH_3OH-C_8H_6O-CO]^-$, 374.0434 $[M-H-CH_3OH-C_8H_6O-CO-CH_3]^-$	$C_{32}H_{22}O_{10}$	GK	Biflavones

19	35.60	565.1160	565.1135	4.4	533.0854 $[M-H-CH_3OH]^-$, 389.0626 $[M-H-CH_3OH-C_8H_6O-CO]^-$, 374.0439 $[M-H-CH_3OH-C_8H_6O-CO-CH_3]^-$	$C_{32}H_{22}O_{10}$	IGG	Biflavones
20	36.02	854.3418	854.3363	6.4	591.2104 $[M+Na-C_{14}H_{16}NO_4]^+$, 286.1034 $[M+Na-C_{31}H_{36}O_{10}]^+$	$C_{45}H_{53}NO_{14}$	CE	Taxoids
21	38.25	834.3089	834.3102	-1.6	549.2018 $[M+Na-C_{16}H_{16}NO_4]^+$, 308.0872 $[M+Na-C_{29}H_{34}O_9]^+$	$C_{45}H_{49}NO_{13}$	7-epi-10-DAT	Taxoids
22	40.17	876.3213	876.3207	0.7	591.2205 $[M+Na-C_{16}H_{15}NO_4]^+$, 533.2152 $[M+Na-C_{16}H_{15}NO_4-CH_3COOH]^+$, 308.0894 $[M+Na-C_{31}H_{37}O_{10}]^+$	$C_{47}H_{51}NO_{14}$	TAXOL	Taxoids
23	40.58	876.3213	876.3207	0.7	591.2208 $[M+Na-C_{16}H_{15}NO_4]^+$, 533.2153 $[M+Na-C_{16}H_{15}NO_4-CH_3COOH]^+$, 308.0893 $[M+Na-C_{31}H_{37}O_{10}]^+$	$C_{47}H_{51}NO_{14}$	7-epi-TAXOL	Taxoids
24	41.07	579.1280	579.1291	-1.9	547.1024 $[M-H-CH_3OH]^-$, 403.0810 $[M-H-C_{10}H_8O_3]^-$, 165.0182 $[M-H-C_{25}H_{18}O_6]^-$	$C_{33}H_{24}O_{10}$	SDN	Biflavones
